# An ecological cascade links climatic variability to avian irruptions and zoonotic salmonellosis outbreaks

**DOI:** 10.1073/pnas.2511209123

**Published:** 2026-01-12

**Authors:** Benjamin A. Tonelli, Casey Youngflesh, Morgan W. Tingley

**Affiliations:** ^a^Department of Ecology and Evolution, University of California Los Angeles, Los Angeles, CA 90095; ^b^Department of Biological Sciences, Clemson University, Clemson, SC 29634

**Keywords:** resource pulse, one health, ecological trap, masting, bird migration

## Abstract

Salmonellosis is a common bacterial infection that can lead to severe illness and death in humans as well as domesticated and wild animals. In bird populations, salmonellosis outbreaks are temporally irregular but occur simultaneously across large spatial extents, frequently leading to widespread avian mortality with occasional spillover to humans. Here, we test the hypothesis that avian salmonellosis epidemics result from a cascade of ecological processes triggered by climate variability in coniferous forests. Building on well-evidenced ecological mechanisms, we find strong evidence for multiyear causal links between temperature cues that drive substantial “boom-bust” cycles in tree cone production across North America, the subsequent irruptions of seed-eating birds, and, finally, the eventual occurrence and severity of avian salmonellosis outbreaks in the United States. By using an integrated, multilevel Bayesian statistical model as a predictive tool to forecast future bird irruptions and disease outbreaks, we demonstrate that our methods can be employed as an early warning system for future epidemics, potentially mitigating outbreak severity among birds and lowering the risk of zoonotic spillover events by encouraging measures such as the temporary cessation of bird feeding ahead of high-risk periods.

## Significance

Predicting and mitigating disease outbreaks is critical for protecting the health of human and wildlife populations. Here, we investigate the ultimate and proximate cause of salmonellosis outbreaks in wild birds from an ecological perspective. Building on well-evidenced mechanisms, we show that climate patterns can alter food availability in coniferous forests, driving bird populations to migrate in high densities, often resulting in salmonellosis epidemics. We then demonstrate how we can use our modeling framework as a predictive tool to create outbreak forecasts that can inform the public about risks to wildlife and human health.

**Fig. 1.**
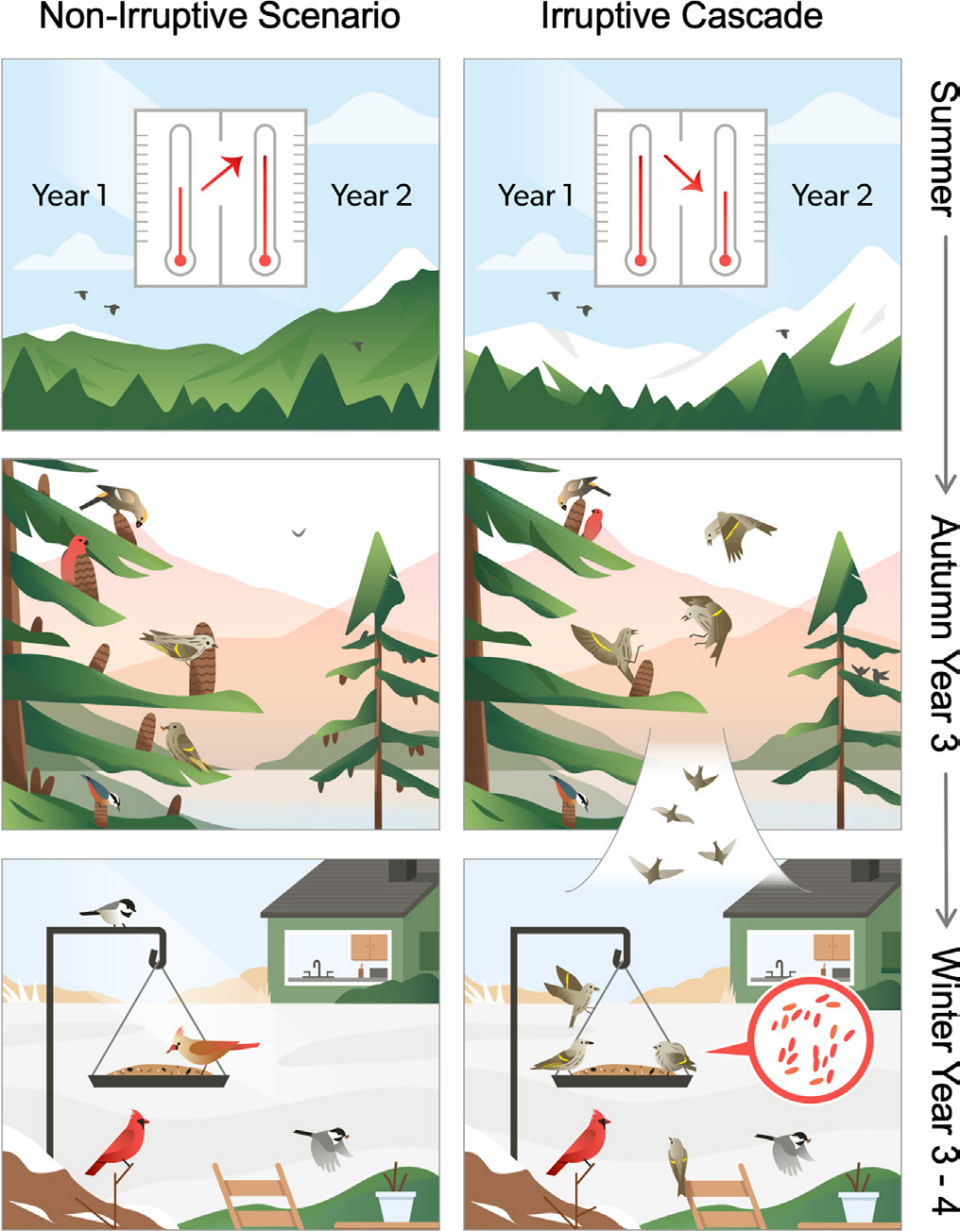
A climate-mediated ecological cascade leads to salmonellosis outbreaks in the United States. Temperature differences in successive summers are associated with differences in tree cone production in high-elevation and high-latitude forests (*Top* row), such that a warm summer followed by a cold summer is associated with a drop in cone production the following year (*Top* *Right* panel). Cone production is negatively associated with facultative bird migrations (irruptions) of eight seed-eating species (*Middle* row), such that when cone numbers are low, birds are more likely to irrupt in large numbers (*Middle* row, *Right* panel). When irruptions of pine siskins (*S. pinus*) occur, more songbirds are often found infected with, or dead from, salmonellosis (*Bottom* *Right* panel).

Understanding the causes of zoonotic outbreaks is a priority for global health and wildlife conservation, yet the ecology of many familiar zoonotic pathogens remains understudied (1, 2). Salmonellosis, a common and widespread disease in wild and domesticated animals, as well as humans, is caused by infection with bacteria from the genus *Salmonella* and spread primarily via a fecal-oral route (3, 4). Periodic salmonellosis outbreaks have been documented in North American songbirds for at least 40 y (4–6), with outbreaks often killing thousands of birds and occasionally spilling over to humans through contact with wild birds and bird feeders (7). Like other zoonoses, the likelihood of salmonellosis outbreaks in birds is determined by a complex combination of factors, including the distribution and movement of host animals, the competence of a given species to harbor and transmit pathogens, and the probability of contact between infected individuals (1, 8). Each of these factors can be influenced by variability in environmental conditions, which might ultimately regulate the probability of outbreak emergence in wildlife and related spillover into human populations (1, 8). Although little is known about the drivers of these periodic avian salmonellosis outbreaks, evidence spread across ecological disciplines suggests a complex cascade of events is responsible (Fig. 1).

Salmonellosis outbreaks among songbirds are especially common in a subset of seed-eating finch species (6). Many of these species share the uncommon life history trait of exhibiting “irruptions,” in which large numbers of individuals sporadically move from high-latitude and high-elevation coniferous forests to more southerly and lower elevation areas in some winters, resulting in substantial localized increases in the number of individual birds in these areas (9). The pine siskin (*Spinus pinus*) is one such finch species that appears to have a particularly strong association with large salmonellosis outbreaks (10), including involvement in a deadly avian epidemic in the winter of 2020–2021 that led to human spillover and subsequent hospitalizations (7). Why pine siskins, in particular, experience high susceptibility to salmonellosis compared to other songbirds remains unclear (6), though their large population size and socially gregarious behavior (10) may contribute. Evidence from other migratory species suggests that the demands of long-distance movements like those that pine siskins undergo can often come at the expense of weakened immune function (11). The rapid dispersal of a high number of social, immunologically stressed irruptive migrants could set the stage for simultaneous epidemics occurring across large spatial extents. These risks may be especially heightened at areas that attract high densities of food-stressed individuals, such as bird feeders (4).

The timing of these irregular, large-scale, facultative movements of irruptive bird species is not random, but rather is driven by the availability of tree seed cones, which function as the primary winter food source for these species (9, 12, 13). When cones are limited, birds flee coniferous forests in large numbers in search of food. Like irruptions, cone production varies dramatically between years but synchronously within years across species and space in a phenomenon known as masting (14). Spatial synchronization of cone production of sympatric tree species acts to alternatively overwhelm (during “mast” years) or starve (during “bust” years) seed predators in a bottom–up trophic process that ultimately regulates population sizes of these animals (14, 15). In turn, these resource pulses in cone production across large spatial scales are driven by shared environmental cues: in particular, the difference between July temperatures 2 y and 1 y prior to cone maturation, a climatological metric referred to as ΔT (16–18). When a warm summer is followed by a cool summer (negative ΔT), cone production in many species is often limited the following year (17) (Fig. 1).

Together, these ecological relationships suggest that interannual temperature variation can result in a higher risk of salmonellosis outbreaks by reducing cone production and triggering bird irruptions. Here, we aim to test the existence and strength of these previously hypothesized relationships linking climate, masting, and bird irruptions, as well as to evaluate the extent to which interannual variability in pine siskin irruptions drives the size of salmonellosis outbreaks among songbirds in North America. To evaluate these hypotheses simultaneously using a single model, we take a hierarchical Bayesian approach that integrates climate, cone production, irruption, and outbreak data from 1980 to 2024. We then illustrate the utility of this model to forecast the probability of avian salmonellosis outbreaks at a regional scale, while accounting for multiple sources of uncertainty and accommodating spatially and temporally heterogenous data availability. As more birds are attracted to growing urban areas with increasing numbers of bird feeders (19), and as climatic conditions become increasingly variable over time, a predictive model like the one presented here can inform wildlife and public health agencies of future outbreak risk across the North American continent and provide a framework for constructing similar tools for other zoonoses.

## Climate Variability Drives Variation in Cone Production.

The first component of our hierarchical model quantifies the relationship between climate variability and cone production. In line with previous work demonstrating the importance of shared environmental cues, we found that cone production across firs, spruces, and hemlocks [families *Abies*, *Picea*, and *Tsuga*, from the MASTREE+ database (20)] was positively associated with an increase in average maximum July temperatures [ΔT, from Daymet (21)] between the 2 y prior to cone maturation (median effect of ΔT on change in cone production relative to the mean, θ = 0.24, 89% CrI = 0.19 to 0.29, *P*(θ > 0) = 1; Eq. **1**). A 3 °C decrease in average temperatures between consecutive years was predicted to nearly halve local cone production compared to consistent summer temperatures between years (median predicted drop = 48.2%, 89% CrI = 41.3 to 56.3%). We also found strong evidence for a previously reported pattern in which mast years are most often followed by bust years, and vice-versa (median 1-y lag effect of cone production, ν = −0.19, 89% CrI = −0.25 to −0.12, p(ν < 0) = 1; Eq. **1**), such that cone production was predicted to be 25% lower following a year in which cone production was 2.5x the average (89% CrI = 16.4 to 32.3%). To evaluate the downstream effects of cone production on bird irruptions, we constructed time series of regional (western and eastern coniferous forests) masting estimates (*SI Appendix*, Fig. S1 and Eq. **5**) using model-estimated relationships between temperature and cone production and the model-estimated spatial structure of masting dynamics (whereby locations in closer proximity are more likely to exhibit similar responses). Regional cone production estimates were highly variable across years, suggesting a high degree of regional spatial synchrony (*SI Appendix*, Fig. S1).

## Limited Cone Production Triggers Bird Irruptions.

**Fig. 2.**
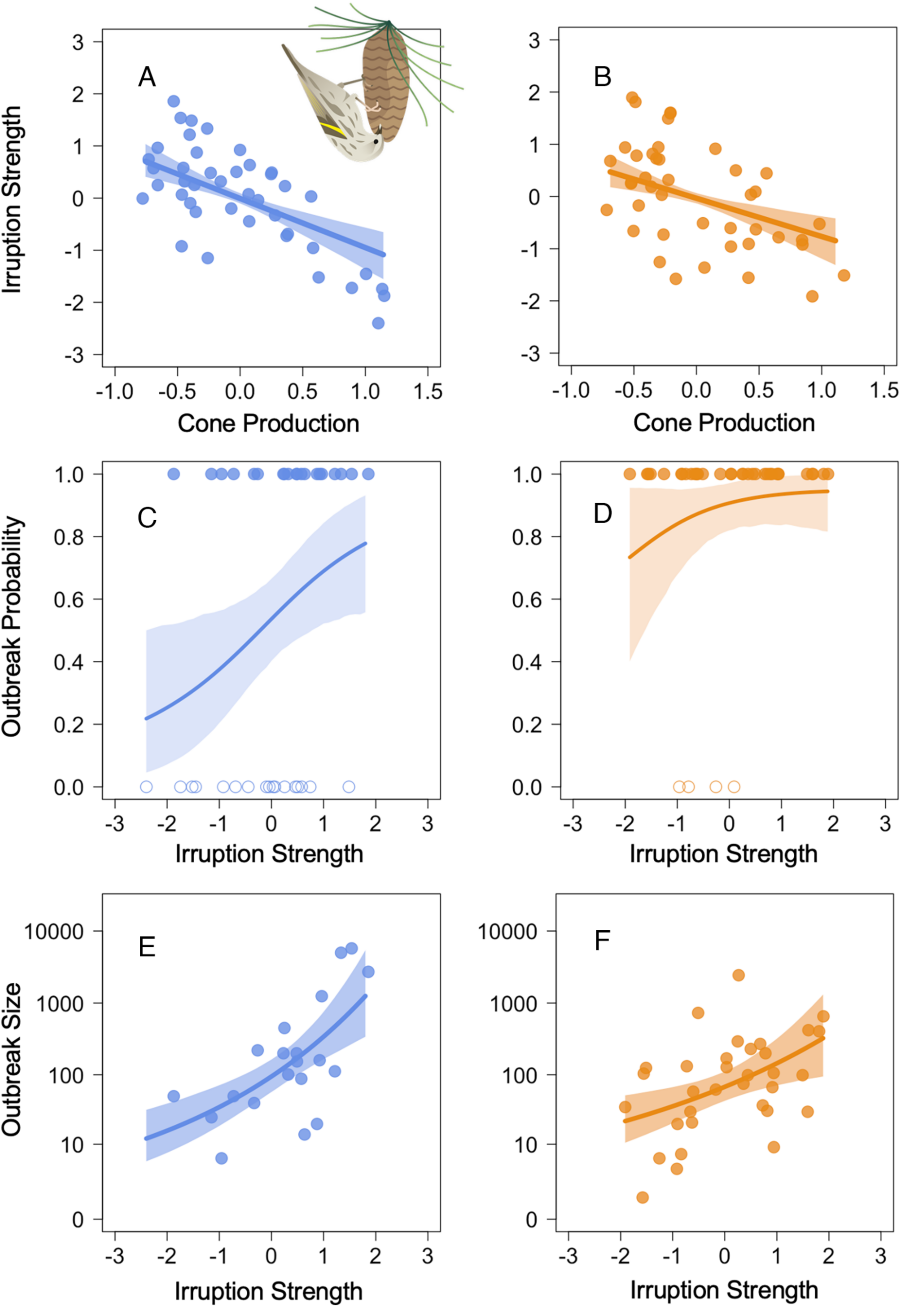
Cone production and salmonellosis outbreaks are linked through pine siskin (*S. pinus*) irruptions. Irruption intensity of pine siskins is negatively associated with regional estimates of cone production (median estimates shown) in both western (*A*) and eastern North America (*B*) with estimates for the species-level effect shown (the solid line indicates mean estimated effect, with 89% credible interval). In turn, the probability of disease outbreaks (solid line, with 89% credible interval) in the western United States is strongly associated with the intensity of pine siskin irruptions (*C*), with a similar effect weakly supported in the eastern region (*D*). Solid points at the top of the plots represent years with outbreaks while open points at the bottom of the plots represent years without outbreaks for (*C*) and (*D*). The number of individual birds reported in salmonellosis outbreaks in both the western (*E*) and eastern (*F*) United States is strongly, positively associated with pine siskin irruption intensity (solid line, with 89% credible intervals).

Concurrent with the estimation of the above relationships, our hierarchical Bayesian model estimated the effect of regional cone production trends on the yearly spatial extent of irruptions of the pine siskin and seven other songbird species across North America using data from the Audubon Christmas Bird Count (CBC) (22). Because of apparent regional differences in irruption and masting timing (23, 24), we estimated relationships within regions separately. In line with previous evidence that irruptions are triggered by winter food scarcity, we found that more spatially widespread (hereafter, “intense”) irruptions were associated with lower regional cone production (median of average cross-species effect of cone production on irruption intensity, western North America: ${\mu_{\omega }}_{1}$ = −0.75, 89% CrI = −1.11 to −0.44, p(${\mu_{\omega }}_{1}$ < 0) = 1; eastern North America: ${\mu_{\omega }}_{2}$ = −0.64, 89% CrI = −1.0 to −0.29, p(${\mu_{\omega }}_{2}$ < 0) = 1; Fig. 2, *SI Appendix*, Fig. S2, and Eq. **6**). We found little to no evidence that irruptions are more likely to occur after a nonirruption year for the typical species in western North America (median 1-y autoregressive effect ${\mu_{\eta }}_{1}$ = −0.10, 89% CrI = −0.25 to 0.05, p(${\mu_{\eta }}_{1}$ < 0) = 0.87; *SI Appendix*, Fig. S2 and Eq. **8**), but strong evidence for a weak effect in eastern North America [median of cross-species effect ${\mu_{\eta }}_{2}$ = −0.24, 89% CrI = −0.37 to −0.12, p(${\mu_{\eta }}_{2}$ < 0) = 1; *SI Appendix*, Fig. S2 and Eq. **8**].

## Bird Irruptions Lead to Salmonellosis Outbreaks.

In contrast to established linkages between climate variability, cone production, and bird irruptions, little is known about the association between bird irruptions and salmonellosis outbreaks. While the pine siskin is known to be involved in large and recent salmonellosis outbreaks (7), we first sought to determine which other irruptive bird species, if any, are important in triggering or amplifying outbreaks. To do this, we calculated the frequency at which irruptive species were reported in outbreak events in the United States using data from WHISPers—a national database of wildlife disease reports (5). Irruptive species were identified in 85% of all winter salmonellosis outbreaks involving passerines in the western United States and 45% of those in the eastern United States between 1987 and 2024. Irruptive species were involved in 79% of the largest outbreaks—measured by the number of dead or sick individuals reported—across regions (≥90th percentile, n = 19).

**Fig. 3.**
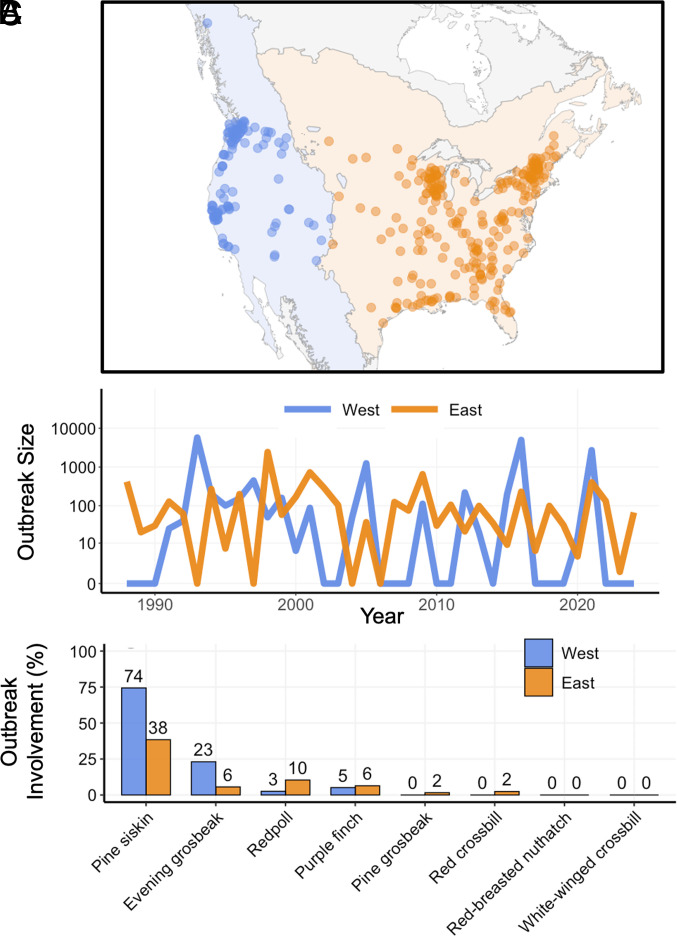
Spatial extent and characteristics of salmonellosis outbreaks among passerines in the United States. Salmonellosis outbreaks were widely reported across the United States in both the western (blue) and eastern (orange) study regions classified via the United States Environmental Protection Agency’s ecoregions (*A*) from 1987 to 2024. The number of individuals reported each winter in regional outbreaks varied interannually (*B*). Irruptive species, especially the pine siskin (*S. pinus*), were reported in a high proportion of outbreaks in both regions (*C*).

Pine siskins were involved in 47% of all outbreak events, nearly twice the rate of the next most reported species (American goldfinch *Spinus tristis*, identified in 27% of outbreaks) and nearly five times the rate of the second most reported irruptive species (evening grosbeak *C. vespertinus*, 10% of outbreaks; Fig. 3). Pine siskins were the most identified species in outbreaks in both the western (74%) and eastern United States (38%, Fig. 3). Based on the close association of pine siskins with historical outbreaks and the species’ extensively documented importance in the outbreak of 2020–2021 (7), we chose to quantify the association between the irruption of pine siskins, in particular, and the frequency and size of salmonellosis outbreaks.

Using our integrated Bayesian hierarchical model, we found that more intense pine siskin irruptions were associated with an increased probability of an outbreak being reported; this linkage was strongly supported in the west [median effect of pine siskin irruption intensity on outbreak likelihood, $\beta_{1}$ = 0.69, 89% CrI = 0.12 to 1.34, p($\beta_{1}$ > 0) = 0.98; Fig. 2 and Eq. **8**] with weaker but still plausible support in the east [median effect of pine siskin irruption intensity on outbreak likelihood, $\beta_{2}$ = 0.60, 89% CrI = −0.28 to 1.60, p($\beta_{2}$ > 0) = 0.86; Fig. 2 and Eq. **8**). In the west, the model-predicted probability of an outbreak rises from 30.3% when pine siskins irruptions are relatively weak (−1.5SD from the mean) to 74.7% when irruptions are more intense (+1.5SD from the mean). In addition to this association between irruption intensity and the likelihood of outbreak reporting, irruption intensity was strongly associated with the size of salmonellosis outbreaks, measured as the total number of sick or dead individuals in each region [western United States: median effect of irruption intensity on outbreak size, $\kappa_{1}$ = 0.25, 89% CrI = 0.13 to 0.35, p($\kappa_{1}$ > 0) = 1; eastern United States: median $\kappa_{2}$ = 0.16, 89% CrI = 0.05 to 0.27, p($\kappa_{2}$ > 0) = 0.99; Fig. 2 and Eq. **9**]. As an example, the model-predicted probability of an outbreak in which at least 500 individual birds (a threshold reached in 7 of 74 regional winters) were reported either ill or dead was 39.3% during an intense irruption year (irruption index +1.5SD from the mean) versus a 0.5% chance during a weak irruption year (−1.5SD from the mean) in the western United States. The effect size was similar in the eastern United States, with a 29.4% chance of a large outbreak during an intense irruption year compared to 2.9% during a weak irruption year.

When considered together, our results demonstrate a clear throughline from summer climate variability in boreal forests to salmonellosis outbreak reports in the United States (Fig. 1). When temperature variability triggers a bust year in cone production, pine siskins tend to exhibit larger irruptions leading to a higher probability and larger predicted size of salmonellosis outbreaks. Given the inherent lag time of roughly 1.5 y between the triggering climate events and the resulting outbreak of salmonellosis among wild birds, we next evaluated the degree to which our statistical model could be used as a forecasting tool for future disease outbreaks.

## Forecasting Salmonellosis Outbreaks.

When outbreaks are detected and reported to wildlife health agencies, officials regularly advise the public to take steps to mitigate risks such as temporarily removing bird feeders (25). Inherently, these warnings from public health officials and related news reports occur only after outbreaks begin. Proactive and accurate early warning systems of wildlife disease risk that are effectively used to engage the public can lead to better mitigation of epidemics compared to reactive approaches (26, 27). In line with guidance from the Centers for Disease Control and Prevention (CDC), reminding the public to regularly clean bird feeders, providing information about how to report and safely dispose of dead birds, and advising the removal of feeders during certain periods, especially when sick or dead birds are observed, are all examples of measures that can reduce epidemic intensity among birds and lower the risk of human spillover (7).

Building on the statistical model highlighted above (and outlined in *SI Appendix*, Fig. S3), we developed a near-term forecasting tool (hereafter referred to as the full forecasting model) that probabilistically estimates the risk of regional disease outbreaks using climate, cone production, and irruption data (*SI Appendix*, Fig. S4). These forecasts have the capacity to inform wildlife health agencies about the likelihood and expected size of potential outbreaks months before they occur. With this information, agencies could then decide to advise members of the public to take steps like removing bird feeders to protect themselves, domesticated animals, and backyard birds from disease risks when the probability of a large outbreak rises above a given threshold.

We evaluated the full forecasting model’s ability to predict the probability and size of outbreaks for upcoming winters given only contemporaneously available information over the last 21 y (half of the study period). To do this, we used a historically informed baseline model that generates probabilistic forecasts based simply on the observed frequency of individuals affected in previous years. Using the continuous ranked probability skill score (CRPSS), a scoring algorithm traditionally used in weather forecasting (28), we found that our full forecasting model outperformed a historically informed baseline model in predicting the annual number of individuals reported in salmonellosis outbreaks (CRPSS = 4.8%, *SI Appendix*, Table S1 and Eqs. **10** and **11**). Here, a score of 100% would be achieved if the model predicts with absolute certainty the exact number of individuals involved in an outbreak, while a score of 0% represents no improvement over the historical baseline model. In addition, we also tested the accuracy of our forecasting models in predicting the occurrence of outbreaks of any size (>0 individuals), large outbreaks (>100 individuals), and severe outbreaks (>500 individuals) using Brier scores (29). In each of these three cases, using the Brier skill score (30), the full forecasting model consistently outperformed the historical baseline models [Brier skill score *P*(>0) = 3%, *P*(>100) = 10.4%, *P*(>500) = 6.1%; *SI Appendix*, Table S2] and vastly outperformed random guessing [i.e., consistent 50% predicted probability of an outbreak of a given size; Brier skill score *P*(>0) = 27%, *P*(>100) = 17%, *P*(>500) = 66.1%; *SI Appendix*, Table S2].

As with all forecasting systems across disciplines, barriers exist for creating more accurate and precise forecasts. For our full forecasting model, one limiting factor to forecast accuracy and precision is the scarcity of up-to-date cone production information. In our historical forecast evaluation, we use cone production data collected in a postpublication database as if it were contemporarily available. However, in practice, the real-time use of this information would depend on close communication with data collectors. In addition to data reporting delays, historical cone production data are spatially and temporally patchy (*SI Appendix*, Fig. S5).

To determine the effect of the availability of cone production data on forecasting skill, we analyzed how forecast accuracy changed when systematically blinded to cone production information but still provided climate data (via a data-blinded forecasting model). Even with this limitation, forecasts of outbreak size with our data-blinded forecasting model showed improvement over historical baseline models (CRPSS = 3.6%, *SI Appendix*, Tables S1 and S2 and Eqs. **10** and **11**), but underperformed the full forecasting model. This gap in predictive accuracy suggests that while forecasts with minimal contemporary cone production data are still useful, increasing the collection of real-time, spatially representative data through citizen science (31) or advances in remote-sensing techniques (32) have the potential to dramatically increase forecast accuracy.

Increasing severity and variability of climate patterns, loss of habitat, altered bird behavior, and changing patterns in supplemental feeding all have the potential to dramatically change the frequency and magnitude of disease outbreaks. Increasing variability in interannual temperatures under climate change may drive greater variation in cone production, although considerable uncertainty in this area remains (18), which may ultimately result in larger irruptions and larger outbreaks. Loss of coniferous forest due to urbanization, deforestation, or wildfires may also increase the risk of spillover events by forcing birds into human-modified landscapes, similar to the patterns seen with Hendra virus infections among Australian bat populations (8). Irruptive species may even adapt their behavior in the long-term in response to anthropogenic and climate-related changes, with unknown effects for disease risk. For example, because pine siskins alter their migratory behavior in direct response to food availability (33), increasing supplemental feeding via bird feeders could lead to the reduction of migratory behavior in this species, potentially either increasing outbreak risk by increasing local density or, alternatively, lowering risk due to less stress-induced immunological effects from lack of food (34). Quantifying the role that complex interactions between the abiotic and biotic environments play in disease dynamics is critical to understanding the ecology of common pathogens and related public health outcomes. This work represents a step in establishing a spatially explicit, process-based predictive framework for salmonellosis outbreaks. In the future, this statistical framework could incorporate greater complexity in ecological processes at more precise spatial scales and be expanded to study how the size of avian outbreaks contributes to the risk of spillover into humans.

## Materials and Methods

### Data and Statistical Model Overview.

We designed a single Bayesian hierarchical model structure that integrates ecological links between climate, cone production, bird irruption, and disease outbreaks, and incorporates information from five independent data sources. A graphical representation of data sources and the study area is provided as *SI Appendix*, Fig. S1, our hypotheses and statistical modeling framework are outlined in *SI Appendix*, Fig. S3, and data availability is displayed as *SI Appendix*, Fig. S5. Our data come from a diverse set of sources: Climate data were derived from Daymet (21), tree masting time series from the MASTREE+ database (20), tree cover data from NASA satellite sensors (35), bird occurrence records from the CBC conducted by the National Audubon Society (22), and disease reports from the Wildlife Health Information Sharing Partnership event reporting system (WHISPers) from the United States Geological Survey (5). Our hierarchical statistical approach models all ecological processes simultaneously, at each level integrating information from these data sources. The model is then used to estimate the strength and direction of the ecological linkages between climate patterns, cone production, irruptions, and disease outbreaks while propagating the estimated uncertainty in these relationships. In general, and for all cases in which a parameter represents a hypothesis being tested, we use broad, uninformative priors centered at zero. We do not incorporate prior knowledge from previous studies into our prior estimates. A full list of priors is available in *SI Appendix*, Table S3. Because climate, cone production, irruptions, and salmonellosis outbreaks appear to show regional differences, our modeling framework is designed to estimate these relationships across two distinct western and eastern regions determined using ecoregion designations from the United States Environmental Protection Agency.

### Climate and Tree Cone Production.

The first level of our model estimates local cone production (alternatively, “masting”) across North American coniferous forests using spatially explicit climate, cone production, and tree cover data. To do this, we first divided the continent using equal-area hexagonal grid cells with roughly 286 km spacing using the R package dggridr (36). We chose this spacing to balance the percentage of cells for which we have cone production measures (aiming to keep this number high via larger cells) while maintaining the spatial representativeness of observed cone production for the cells to which they belong (via smaller cells). We then filtered to include cells where at least 25% of land area was within one of three target Environmental Protection Agency level-one ecoregions dominated by conifer forests (i.e., Northwestern Forested Mountains, Marine West Coast Forests, or Northern Forests). Next, we filtered to only include cells that were overwhelmingly land (>95%). Based on the ecoregion most represented in each cell, we then categorized cells as either representing western forests (Northwestern Forested Mountains and Marine West Coasts Forests ecoregions) or eastern forests (Northern Forests ecoregion; *SI Appendix*, Fig. S1). We then extracted daily maximum temperatures during July from Daymet (21) for each of these cells for the period 1980–2023. We calculated the difference between the average maximum daily July temperature in consecutive 2-y periods (ΔT) for each cell (i.e., average temperature in year t minus average temperature in year t-1).

We then estimated cone production of tree species with 2-y reproductive cycles for each cell and year from 1982 to 2024. To do this, we incorporated empirically measured cone production data from MASTREE+, a database that compiles reproductive output information from published studies (20). We filtered the MASTREE dataset to the genera with 2-y reproductive cycles: *Abies* (firs), *Picea* (spruces), and *Tsuga* (hemlocks). We focused on these species due to their shared reproductive timeline and the broad spatial distribution of available data. We excluded pines of the genus *Pinus* from our analysis due to poor spatial coverage, more complex associations between cone production and temperature differences during cone development, and lower interannual variation in masting (17). Because of the known cross-species temporal synchronicity of cone production (37), and the dominance of the three included genera across much of the study region (38), we expect data included here to be a reliable proxy for local cone production. In the future, the incorporation of cone production data from *Pinus* species could likely improve the explanatory power and forecasting skill of our model. In total, our masting dataset compiled 1,821 data points from 131 unique time series from at least eight species (some trees were identified only to genus). For each time series, we standardized cone production data by dividing by the mean cone production of that time series, such that a transformed value of one represents the mean observed cone production for a given cell. To model local masting with a gamma distribution, we added a small scalar (0.001) to all local masting estimates to avoid zeros at this step but back transformed these values downstream (i.e., for use as the covariate $m_{c,t-1}$ and estimates of regional masting trends, explained below). When cell-year combinations had one or more empirical measure of cone production we averaged standardized values across time series. After compiling, data included in the model totaled 546 data points from 38 cells. Cone production data are incomplete—representing between 0% and 27% of cells in each year (*SI Appendix*, Fig. S5). To account for this, we estimated local masting in cells and years without data in our Bayesian framework as unobserved states.

Following (17), we modeled relative local cone production (m) at each site (index *c*) as a function of variables previously reported to have an effect on tree reproductive indices: ΔT and reproductive output from the previous year ($m_{t-1}$) using the linear predictor of cone production (ξ):

[1] $$\xi_{c,t}=\alpha_{t}+\nu *m_{c,t-1}+\theta *\Delta T_{c,t}+\phi_{c,t}*\sigma_{\phi },$$

where α represents a year-specific intercept, θ is the effect of ΔT on masting, and the terms $\phi_{c,t}$ and $\sigma_{\phi }$ constitute a conditional autoregressive component (39) used to model the spatial autocorrelation in local masting between neighboring cells, where $\phi_{c,t}$ is the spatial effect and $\sigma_{\phi }$ is the scale of that effect across all years. The term ν represents the temporal autoregressive effect of masting in the previous year. We chose a spatial modeling approach that included estimating masting in unknown areas to robustly account for uncertainty at the regional level (i.e., resulting from missing masting information) while incorporating model-estimated effects of spatial autocorrelation and the effects of ΔT on masting. Because many unmeasured masting values are estimated by the model, to ensure identifiability we set a constraint on the mean masting value across all years and cells such that the average masting value is constrained to be close to 1, similar to the soft sum-to-zero method used to constrain spatial effects (39). Via a restrictive prior set on the mean of all masting values across all years, this prevents the model from estimating biologically unrealistic average masting values across the entire study period and potentially biasing coefficient estimates.

We modeled local masting m from a gamma distribution with the shape parameter υ:

[2] $$m_{c,t}∼Gamma\left(\upsilon ,\frac{\upsilon }{e^{lp_{c,t}}}\right).$$

We explored using a log-normal distribution but found a gamma distribution was a better fit for these data. We model local masting in the year immediately preceding the first year as an unknown state. To generate realistic cone production patterns in this year, local masting values are drawn from the same distribution described above with all variables in the linear predictor set to the mean observed over the study period. Additionally, to constrain the regional masting indices to realistic values in this year, the mean observed masting across the entire study area is drawn from a distribution determined by the model-estimated yearly mean and SD in masting across all years.

Spatial cells used in this analysis contain varying amounts of forested area. For this analysis, we assumed the contribution of each cell to regional cone production was proportional to the amount of forested area within that cell. To calculate total relative cone production across each region (rm), we took the yearly sum of local cone production across all cells weighted by the percent tree canopy cover (estimated in 2000) of each cell, FC:

[3] $${\mathrm{rm}}_{r,t}=\frac{\sum_{c}\left(m_{c,t}*X_{r,c}*FC_{c}\right)}{\sum_{c}\left(X_{r,c}*FC_{c}\right)}.$$

To assign each region to a cell, we used X which encodes (using zeros and ones) whether each cell (*c*) belongs to a given region (*r*). As such, rm represents a yearly regional estimate of cone production relative to mean average regional masting. These regional masting estimates are then standardized as RM:

[4] $${\mathrm{RM}}_{r,t}=rm_{r,t}-\frac{\sum_{t}(rm_{r,t})}{N_{t}}.$$

By subtracting the average of all regional masting estimates (where $N_{t}$ is the number of years) from each yearly regional masting estimate.

### Tree Cone Production and Regional Irruptions.

Using standardized, regional cone production estimates we then model the relationship between cone production and bird irruptions. To construct bird irruption time series, we requested CBC data from the National Audubon Society covering the United States and Canada from the inception of the program to the winter ending in 2024 (22). CBC is a long-term monitoring program detailing the winter occurrence of birds using compiled accounts of species abundance via volunteer birdwatcher checklists collected in December or January within 24 km diameter circles. Although we were primarily interested in the irruption dynamics of the pine siskin (*S. pinus*), we chose to model the irruptions of seven other species known to show irruptive dynamics as there may be some synchronicity of these irruptions: red-breasted nuthatch (*Sitta canadensis*), evening grosbeak (*Coccothraustes vespertinus*), pine grosbeak (*Pinicola enucleator*), purple finch (*Haemorhous purpureus*), red crossbill (*Loxia curvirostra*), white-winged crossbill (*Loxia leucoptera*), and redpoll (*Acanthis flammea*). Due to the recent reclassification of two former North American redpolls as a single species (40), we lumped all CBC records coded as hoary and common redpoll into a single species: redpoll. Because irruptions of multiple species occur in the same winter, the irruption dynamics of other species could provide additional information about the ecological relationships modeled here and add information to predictions of future pine siskin irruptions. Other North American bird species are known to exhibit irruptive behavior, including the pinyon jay (*Gymnorhinus cyanocephalus*), a pinyon pine specialist of the American Southwest, and the northern hawk owl (*Surnia ulula*), a mammalian and avian predator of boreal forests; however, we chose to focus on the eight species included here due to both their shared boreal distributions and similar winter diet.

For each species in both eastern and western North America, we calculated the spatial extent of irruptions for each year. To do this, we calculated the percent of unique spatial cells that a given species, including all subspecies, were observed in for each region during each winter season. Similar to the method used to assign cells to coniferous forest regions described above, we first created a grid across North America of equal-area cells with roughly 95 km spacing using the package dggridr (36). We used smaller grid cells at this level due to greatly increased data availability (*SI Appendix*, Fig. S1). Each cell was assigned to either the eastern or western North America region based on the EPA level one ecoregion they best represented as a proportion of total area. Because irruptions can result in species expanding their range both within and outside of coniferous forests, at this level we expand the eastern and western regions to encompass most of North America (*SI Appendix*, Fig. S1).

Because we were primarily interested in short-term variability in irruptions, and to control for variable sampling effort over time (both in the number and locations of CBC circles being sampled, and the number of observers per cell), we detrended irruption time-series (i.e., the percentage of cells a given species was observed in) using smoothed splines (df = 8). We then truncated the data to exclude all years prior to when we had relevant cone production data (prior to the winter ending in 1983). We then normalized the time series for each species. Together, this resulted in normalized, detrended, regional- and species-specific irruption time series, *I* (*SI Appendix*, Fig. S3). Because of this normalization and detrending, we are not accounting for the potential effects of long-term trends in pine siskin irruptions on disease outbreak probability or severity.

We modeled the relationship between irruptions (*I*) of each species, i, and regional masting indices (*RM*) using a multivariate normal distribution to account for synchronicity in irruptions across species with a region:

[5] $$I_{r,i,t}∼MVN\left(\gamma_{r}+\omega_{r,i}*{\mathrm{RM}}_{r,t}+\eta_{r,i}*I_{r,i,t-1},\Sigma_{r}\right),$$

where γ represents the intercept and ω represents the effect of regional masting on irruptions, and η represents the autoregressive effect of irruptions in the previous winter on irruptions in the current winter. The covariance of irruptions across species, after accounting for shared effects of predictor variables, is represented by 8 × 8, region-specific covariance matrices, Σ_*r*_. We chose to use a multivariate normal rather than a random effects structure to allow the model to predict future irruption states of multiple species using information from previous years via estimated species-level autoregressive effects.

Species-level effects of regional masting on irruption size and temporal autoregressive effects were modeled hierarchically:

[6] $$\omega_{r,i}∼N\left(\mu_{\omega_{r}},\sigma_{\omega_{r}}\right),$$

[7] $$\eta_{r,i}∼N\left(\mu_{\eta_{r}},\sigma_{\eta_{r}}\right),$$

where $\mu_{\omega_{r}}$ represents the cross-species effect of cone production on irruptions, and $\mu_{\eta_{r}}$ represents the cross-species effect of irruptions in the previous year.

### Irruptions and Disease Outbreaks.

In the last level of our model, we model salmonellosis outbreaks as a function of pine siskin irruptions. To do this, we downloaded disease event data from October–April for winters ending in 1988 to 2024 from the WHISPers database and filtered to reports with salmonellosis listed as a possible diagnosis from the United States (5). The WHISPers database includes opportunistically collected disease reports sourced from approved agencies, including local government wildlife departments. After filtering, we further narrowed this dataset to all reports involving at least one passerine species, effectively excluding reports that consisted exclusively of waterfowl. We chose to exclude disease reports among waterfowl because the disease ecology of salmonellosis likely differs substantially between these two groups. We then classified disease reports by winter season using the midpoint of the reported event dates, except in a single case where we used the start of the event date because the end date was missing. After filtering, we georeferenced the locations of each event—either a list of counties and/or states—to determine whether the outbreaks occurred in the eastern or western region using the geographical center of each county or state derived from maps from the National Weather Service (available via https://www.weather.gov/gis/Counties). We constructed regional salmonellosis time series by calculating the total number of individuals from all species reported as dead or sick in each region during each winter season. We chose aggregation at the regional level to mitigate the potential effects of spatial gaps in disease reporting that may result in false absences (e.g., a local wildlife health department does not consistently report disease data to WHISPers). Although most species reported were birds, other animals like domestic cats (*Felis catus*, n = 3) and one unlucky muskrat (*Ondatra zibethicus*, n = 1) were reported as being involved in winter salmonellosis outbreaks. Like any opportunistic dataset, it is likely that disease events are underreported. However, we did not observe any clear linear trends in outbreak frequency or severity during the study period that might suggest a change in reporting frequency over the study period (Fig. 3). Because pine siskin irruption time series are detrended, any correlation between reported outbreaks and irruptions are the result of interannual variation rather than long-term trends.

We then modeled the log-transformed number of individuals (adding one to all values to avoid taking the log of zero) reported in all regional disease outbreaks (D) using a hurdle model with a gamma distribution to account for the overrepresentation of zeros in regional outbreak time series (i.e., years in which no outbreaks were detected). We considered using a zero-inflated Poisson distribution using the nontransformed data but found it a worse fit for the extremely right-skewed distribution of outbreak sizes. The first step of this model estimates the effect of irruptions on the probability of an outbreak of any size being reported as a function of pine siskin irruptions, $I_{r,1,t}$:

[8] $$P\left(D_{r,t}\right)=\{\begin{array}{ll} \theta & if\;D_{r,t}=0\\ \left(1-\theta \right)*Gamma\left(\lambda_{r},\frac{\lambda_{r}}{e^{\zeta_{r}+\kappa_{r}*I_{r,1,t}}}\right) & if\;D_{r,t}>0 \end{array},$$

[9] $$\theta =1-\left(\frac{1}{1+e^{\psi_{r}+\beta_{r}*I_{r,1,t}}}\right),$$

where 1-θ is the probability of the occurrence of an outbreak of any size, ψ is the intercept term, and β is the effect of pine siskin irruptions on the likelihood of a regional outbreak. If an outbreak does occur, the outbreak size (measured as the total number of individuals reported sick or dead in each region) is modeled as a function of pine siskin irruption size where ζ is the intercept, κ is the effect of pine siskin irruptions on outbreak size, and λ is the shape parameter.

### Model Fit and Reporting.

We fit our statistical models, including the forecasting models described below, using the package rstan (41). For the hypothesis testing model, we ran our model with 8,000 iterations total, 2,000 warm-up iterations, and a thin factor of two. For the forecasting models (see below), we ran our model with 8,000 iterations total, 2,000 warm-up iterations, and a thin factor of two except for the two least informed models which needed more iterations (14,000) to pass model checks. We implemented thinning due to the excessive space requirements (~1.5 GB for each of the 43 models fit). All model parameters were checked for convergence, with a $\hat{R}\leq$ 1.03 and effective sample sizes ≥ 400, and for problematic posterior geometry indicated via divergences or energy warnings. The posterior predictive overlap was assessed using *MCMCvis* (42) and is reported in *SI Appendix*, Table S3 and Dataset S1. For our hypothesis testing model, we also checked for problematic parameter correlations via the package *shinystan* (43), and observed no issues.

In reporting our results, we use the “language of evidence” framework to avoid traditional and problematic cutoffs for statistical significance (44). We report 89% credible intervals in line with (45), and the proportion of the posterior that is greater than (p(PARAMETER>0) or less (p(PARAMETER<0) than zero for each parameter.

### Forecast Generation and Analysis.

We used our statistical model to predict future conditions by modeling the upcoming state of cone production, irruptions, and outbreaks as unknown states. Functionally, the model predicts future cone production using known climate data and estimates of local masting from previous years, as well as a yearly intercept value $\alpha_{t}$ drawn from the model-estimated distribution $N(\mu_{\alpha },\sigma_{\alpha })$.

To determine whether our hypothesis-testing model was effective at forecasting, we fit a series of independent, identically structured statistical models (hereafter, “full forecasting model”) using 21 different historical datasets, each with successively longer time series. This predictive evaluation simulates how the model would have performed if it was used to make predictions starting 21 y ago (starting at the midpoint of the study period), updated each year with new data, and then evaluated the following year using observed data. Although we focused on predicting upcoming outbreaks in the near-term (with an approximate 6-mo lead time), our model could be used to predict outbreaks farther into the future (likely with declining efficacy). In addition, we also trained models using the same methodology, except that models were blinded to contemporary cone production information (hereafter, “blinded forecasting model”).

To assess the value of the full and blinded forecasting models, we compared probabilistic model-predicted irruption and outbreak size distributions to those generated via a probabilistic historical null model (“historical model”). The historical model simply assumes that the probability of an irruption and outbreak of a given size is equivalent to the frequency at which those events have occurred in previous years (i.e., no predictor variables are used). Using a measure of probabilistic forecasts called the continuous ranked probability score, or CRPS (46), using the r package *scoringRules* (47), we scored each forecast of regional irruptions and disease outbreak size for the historical, blinded, and full models. The CRPS score penalizes forecasts both for being imprecise (i.e., uncertain) and for being inaccurate (i.e., biased). We then calculated the CRPSS of the full and blinded model by comparing the sum of scores across the 21 y of forecasts to those generated by the historical model (48):

[10] $${\mathrm{CRPSS}}_{\mathrm{full}}=1-\frac{\sum {\mathrm{CRPS}}_{\mathrm{full}}}{\sum {\mathrm{CRPS}}_{\mathrm{historical}}},$$

[11] $${\mathrm{CRPSS}}_{\mathrm{blinded}}=1-\frac{\sum {\mathrm{CRPS}}_{\mathrm{blinded}}}{\sum {\mathrm{CRPS}}_{\mathrm{historical}}}.$$

For reporting purposes, we then convert CRPSS to a percentage (*SI Appendix*, Table S1). Skill scores provide a measure of the relative accuracy and precision of a probabilistic forecast compared to an existing benchmark, with a positive value representing an improvement. To represent the performance of our model-generated forecasts, we provide a graphical representation of the model-estimated likelihoods of outbreak of different sizes over the last 21 y (*SI Appendix*, Fig. S4). Because some parties may be interested in the predictive accuracy of our forecasting model to predict outbreaks of various sizes—for example, an agency may only want to send a warning to the public if the probability of a severe outbreak is high—we provide an assessment of the accuracy of the historical baseline model, the blinded model, and the full forecasting model using Brier scores (29). We compare the performance of each model using the Brier skill score (30) and report these results in *SI Appendix*, Table S2.

## Supplementary Material

Appendix 01 (PDF)

Dataset S01 (PDF)

## Data Availability

All code needed to conduct this analysis and reproduce all figures is available on GitHub (https://github.com/bentonelli/Climate_to_Salmonella_PNAS) (49) and archived on Zenodo (https://doi.org/10.5281/zenodo.17914985) (50). Our analysis relies on data from third parties, and we do not have permission to publish these datasets in their original form. We provide instructions for how interested parties can request and access this data in the README file.
