# Supplementary material for: An ecological cascade links climatic variability to avian irruptions and zoonotic salmonellosis outbreaks
Source: Proc Natl Acad Sci U S A. 2026 Jan 12;123(3):e2511209123. doi: 10.1073/pnas.2511209123 (PMC12818414; doi:10.1073/pnas.2511209123)
Supplement: Supplementary file 1 — Appendix 01 (PDF) [file pnas.2511209123.sapp.pdf]

PNAS

Supporting Information for

An ecological cascade links climatic variability to avian irruptions and zoonotic salmonellosis outbreaks

Benjamin A. Tonelli, Casey Youngflesh, Morgan W. Tingley

Benjamin Tonelli
Email: btonelli@ucla.edu

This PDF file includes:

Figures S1 to S5
Tables S1 to S3

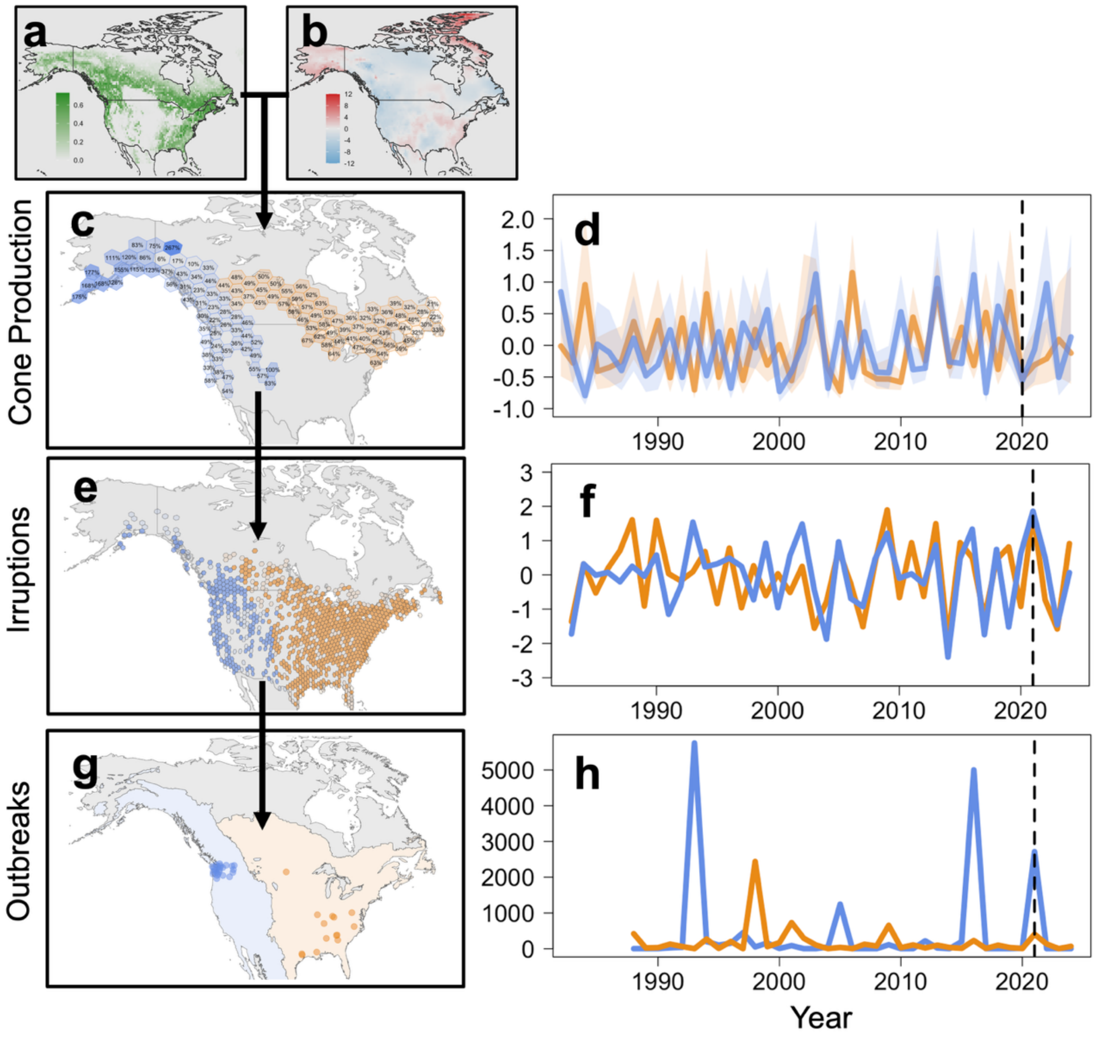

Figure S1. Methodological overview. Our approach utilizes data across ecological levels to model the relationship between climate, cone production, irruptions, and salmonellosis outbreaks. Here, we display data pertinent to the irruption and salmonellosis outbreak observed in the winter of 2020-2021 in the left column (a,b,c,e,g), and time series for the entire study period in the right column (d,f,h). Tree cover data (a) and temperature information (b) are used to estimate cone production at a local (c) and regional (d) scale. Regional cone production indices (d) are then compared to regional irruption time series of the pine siskin (*Spinus pinus*, f) derived from presence/absence records of the species

across the United States and Canada (e). Finally, the number of individuals reported in regional salmonellosis outbreaks (h) are modeled as a function of pine siskin irruption intensity.

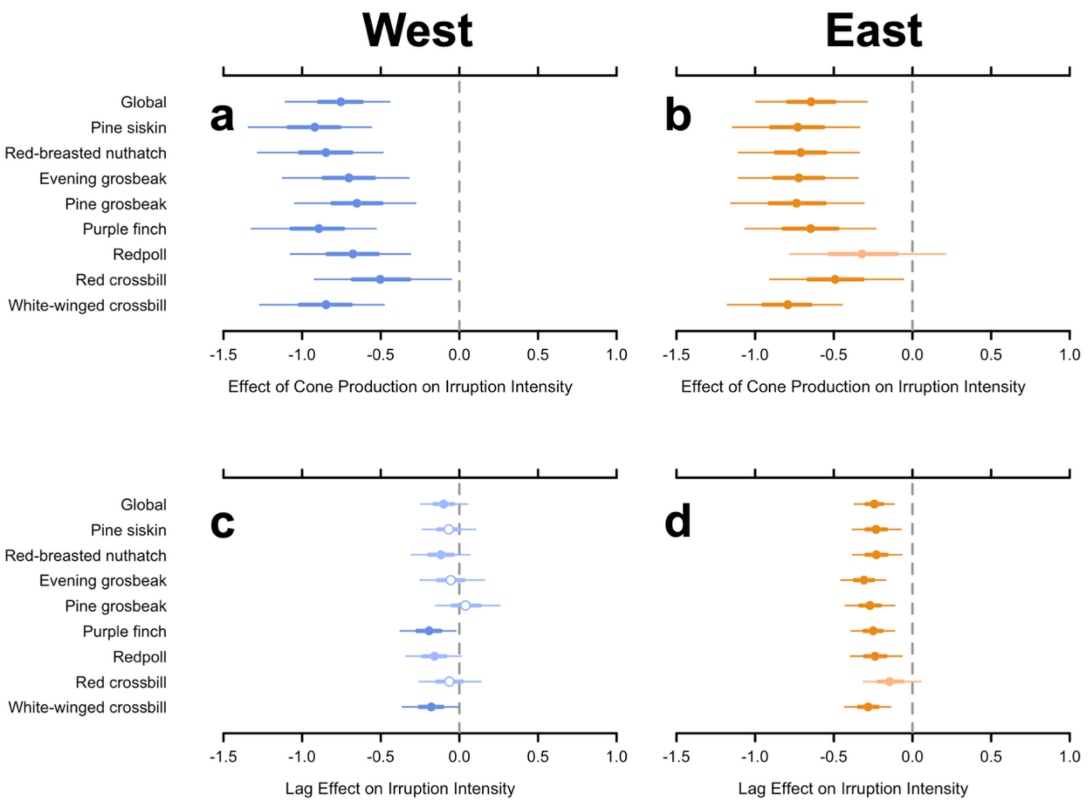

Figure S2. Model-estimated global and species-level effects of cone production (a,b) and lag effects (c,d) on irruption intensity in the western (left column) and eastern region (right column). Dots represent the median estimated effect, with thick lines representing the 50% credible interval (CrI) and thin lines representing the 89% CrI. When 89% CrIs do not overlap zero, effects are represented with dark colors. When the 89% CrI crosses zero, colors are muted. When the 50% CrI overlaps zero, the median effect is represented with an open circle.

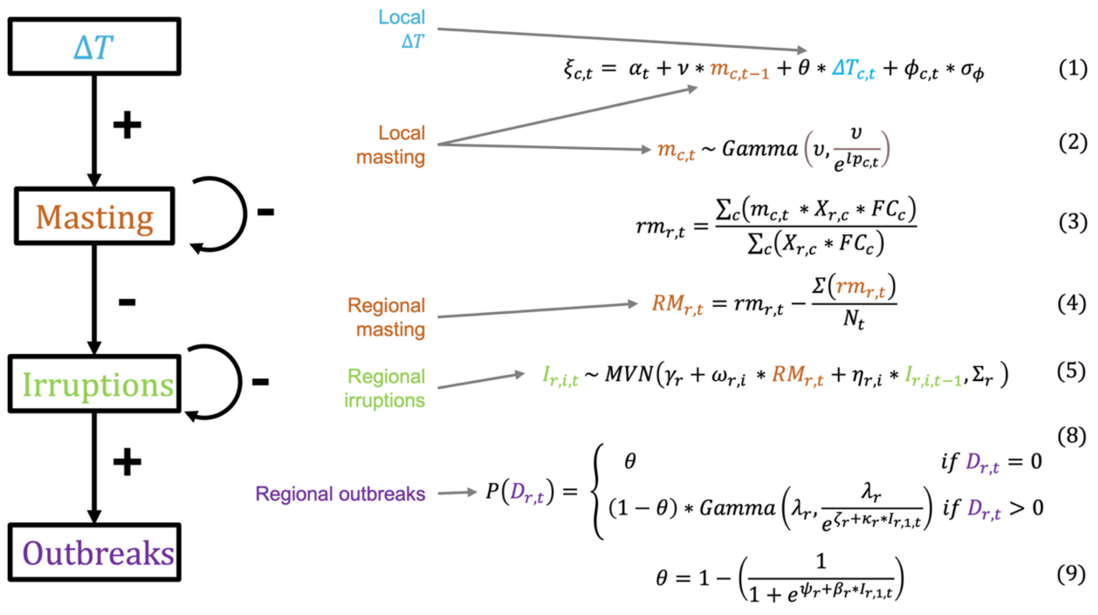

Figure S3. Overview of statistical model structure and hypothesized ecological relationships. Climate variability (*ΔT*) is hypothesized to be positively associated with cone production (alternatively, masting) at the local level (Eq. 1 & 2). At regional scales, cone production indices (Eq. 3,5) are hypothesized to be negatively associated with irruptions (Eq. 6). Lastly, we hypothesized that irruptions are positively associated with outbreaks such that outbreaks are both more likely and larger during intense irruption years (Eq. 9 & 10). Equation numbering corresponds to their appearance in the methods section.

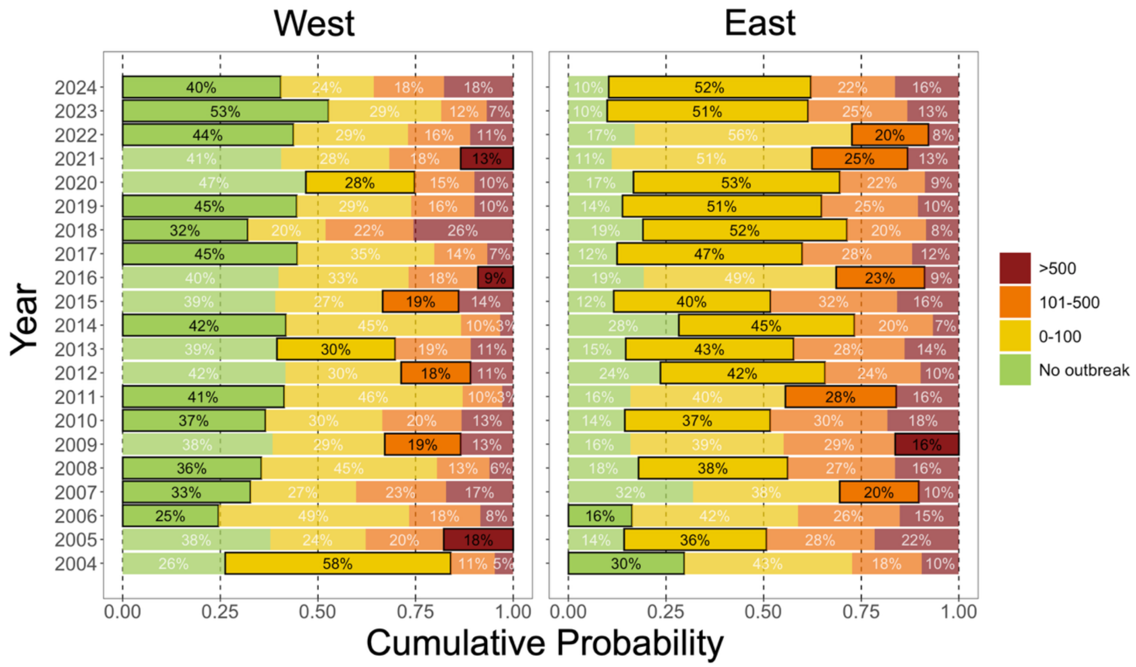

Figure S4. Forecasts of regional outbreak risk can be generated each year for dissemination for wildlife and public health authorities. Model-generated regional risk assessments for winter seasons ending 2004-2024 are shown, with bin width indicating the model-estimated probability of no outbreak (green) and outbreaks of increasing sizes (yellow, orange, red). The observed outbreak size is indicated via darker colored bins with black borders.

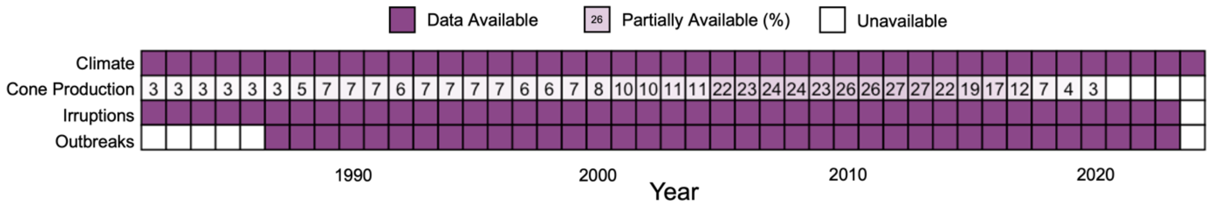

Figure S5. Availability of data by year across ecological levels. Climate, irruption, and outbreak data are available in most years, while cone production data (represented as percentage of all cells) is temporally and spatially sparse, with a maximum of 27% of modeled cells reporting data in a single year.

Table S1: Predictions generated using our full forecasting model outperformed those generated by a historical baseline model for regional outbreak size and irruption intensity. Probabilistic forecast quality was assessed via the continuous ranked probability score (CRPS), a metric commonly used in weather forecasting, with lower numbers indicating better scores. To compare our forecasting models under various data availability conditions, we used the continuous ranked probability skill score (CRPSS) to assess the relative skill of full forecasting and blinded forecasting models compared to the historical baseline model (see Eq. 11-12). Opposite to the scoring with CRPS, a positive CRPSS score represents improvement, with a value of 100% represents the maximum improvement possible (i.e. 100% certainty of the exact value of the future state). For example, a perfect score forecasting model would indicate that each year the generated forecast predicts with 100% certainty the exact number of individuals reported in that outbreak. Our blinded forecasting model (which was not provided contemporary cone production data), outperformed the historical baseline model for predicting pine siskin irruptions and disease outbreak size but underperformed compared to the full forecasting model.

		Forecasting Model		
		Historical Baseline	Blinded	Full
Outbreak Size	Model Score (CRPS)	58.9	56.8	56.1
	**Improvement Score (CRPSS)**	–	**3.6%**	**4.8%**
Irruption Intensity	Model Score (CRPS)	30.9	27.1	25.4
	**Improvement Score (CRPSS)**	–	**12.1%**	**17.7%**

Table S2. Assessment of model predictive accuracy using Brier scores (BS) and relative accuracy across models using Brier skill scores (BSS). Brier scores are reported for the predicted probability of regional outbreaks of various sizes (greater than 0, 100, and 500 individuals), and the relative performance of each model was compared using the Brier skill score. To aid in interpretation, we also report the Brier score resulting from prediction with consistent 50% probability of an outbreak of a given size (reported here as "random"), and the Brier skill score of each forecasting model in comparison. Across each of the three outbreak size thresholds tested here, the full forecasting model performed best, followed by the blinded model and the historical baseline model.

			Forecasting Model			
			Random	Historical Baseline	Blinded	Full
Outbreak Size	>0	Model Score (BS)	0.25	0.188	0.184	0.183
		**Improvement Score Over Random (BSS)**	–	**24.7%**	**26.3%**	**27%**
		**Improvement Score Over Historical (BSS)**	–	–	**2.1%**	**3%**
	>100	Model Score (BS)	0.25	0.232	0.214	0.207
		**Improvement Score Over Random (BSS)**	–	**7.37%**	**14.3%**	**17%**
		**Improvement Score Over Historical (BSS)**	–	–	**7.5%**	**10.4%**
	>500	Model Score (BS)	0.25	**0.09**	**0.089**	**0.085**
		**Improvement Score Over Random (BSS)**	–	**63.9%**	**64.6%**	**66.1%**
		**Improvement Score Over Historical (BSS)**	–	–	**1.9%**	**6.1%**

Table S3. Prior specification and posterior predictive overlap, grouped by model level. Indexes are reported when prior applies to more than parameter (i.e. unexplained variance of species-level irruptions, σ_1 and σ_2). Note that gamma distribution notation describes alternative parameterization with shape and rate.

	Parameter Name	Index	Prior	Posterior Predictive Overlap (%)
Cone production	μ_α		$N(0,4)$	6.4
	σ_α		$Gamma(2,2)$	23.1
	ν		$N(0,1)$	9.5
	θ		$N(0,1)$	7.2
	υ		$N(1,1)$	11.1
	σ_ϕ		$N(1,0.5)$	22.7
Bird irruptions	γ_1		$N(0,1)$	14.0
	μ_{ω_1}		$N(0,3)$	15.2
	σ_{ω_1}		$Gamma(3,3)$	30.4
	μ_{η_1}		$N(0,3)$	8.4
	σ_{η_1}		$Gamma(3,3)$	15.4
	γ_2		$N(0,1)$	14.2
	μ_{ω_2}		$N(0,3)$	15.9
	σ_{ω_2}		$Gamma(3,3)$	31.8
	μ_{η_2}		$N(0,3)$	7.1
	σ_{η_2}		$Gamma(3,3)$	10.1
	σ_1	1-8	$Gamma(3,2)$	23.9 to 29.9
	σ_2	1-8	$Gamma(3,2)$	21.8 to 29.3
Outbreaks	ψ_1		$N(0,5)$	14.8
	ψ_2		$N(0,5)$	22.1
	β_1		$N(0,3)$	24.2
	β_2		$N(0,3)$	34.2
	λ_1		$Exponential(0.05)$	44.1
	λ_2		$Exponential(0.05)$	28.3
	ζ_1		$N(1,2)$	8.4
	ζ_2		$N(1,2)$	8.3
	κ_1		$N(0,3)$	6.1
	κ_2		$N(0,3)$	5.8
