# Supplementary material for: An ecological cascade links climatic variability to avian irruptions and zoonotic salmonellosis outbreaks
Source: Proc Natl Acad Sci U S A. 2026 Jan 12;123(3):e2511209123. doi: 10.1073/pnas.2511209123 (PMC12818414; doi:10.1073/pnas.2511209123)
Supplement: Supplementary file 2 — Dataset S01 (PDF) [file pnas.2511209123.sd01.pdf]

PNAS

Supporting Information for

An ecological cascade links climatic variability to avian irruptions and zoonotic salmonellosis outbreaks

Benjamin A. Tonelli, Casey Youngflesh, Morgan W. Tingley

Benjamin Tonelli
Email: btonelli@ucla.edu

This PDF file includes:

Dataset 1

Dataset 1: Graphical representation of posterior predictive overlap for model parameters of the main hypothesis-testing model.

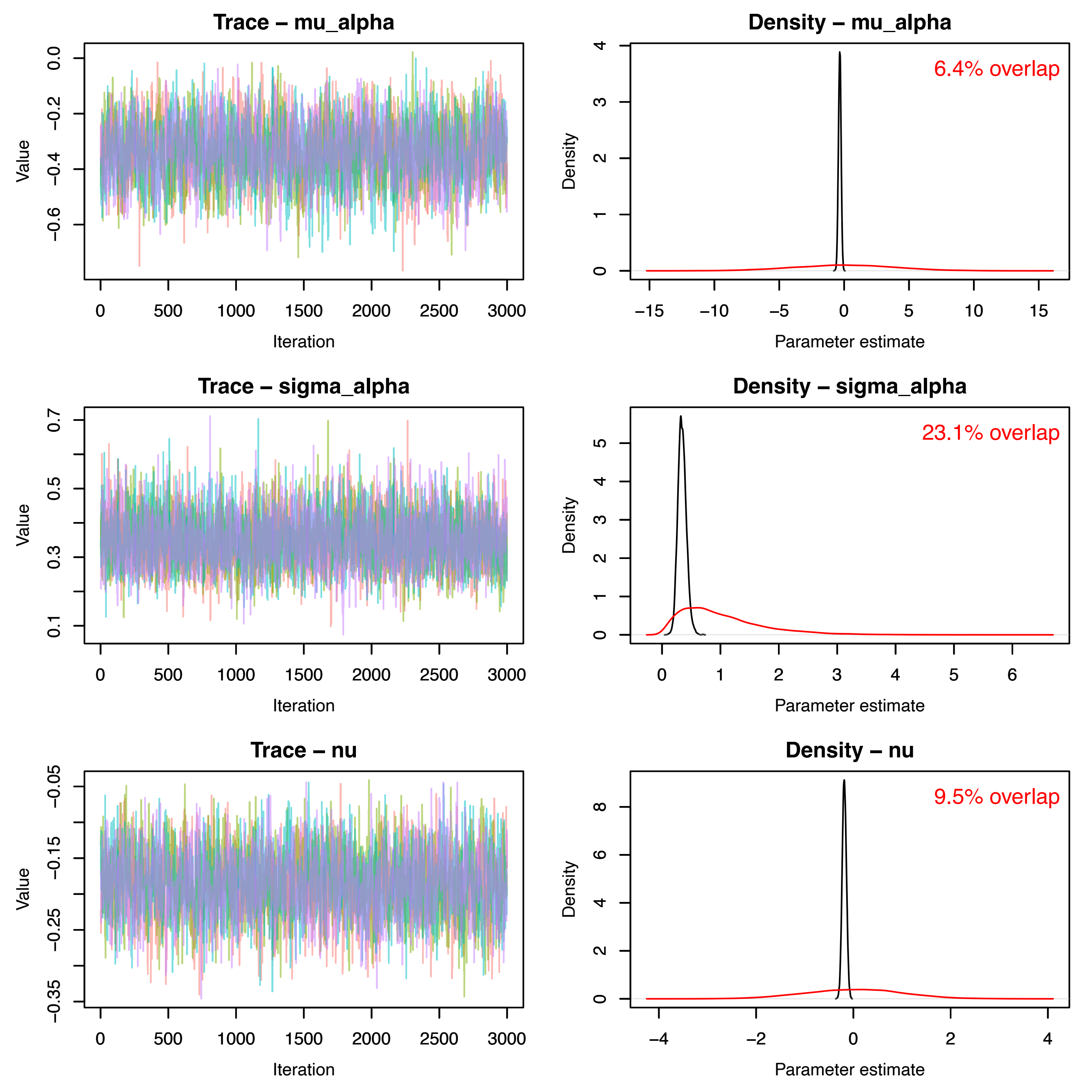

Trace – mu_alpha
Value
Iteration
Density – mu_alpha
6.4% overlap
Density
Parameter estimate
Trace – sigma_alpha
Value
Iteration
Density – sigma_alpha
23.1% overlap
Density
Parameter estimate
Trace – nu
Value
Iteration
Density – nu
9.5% overlap
Density
Parameter estimate

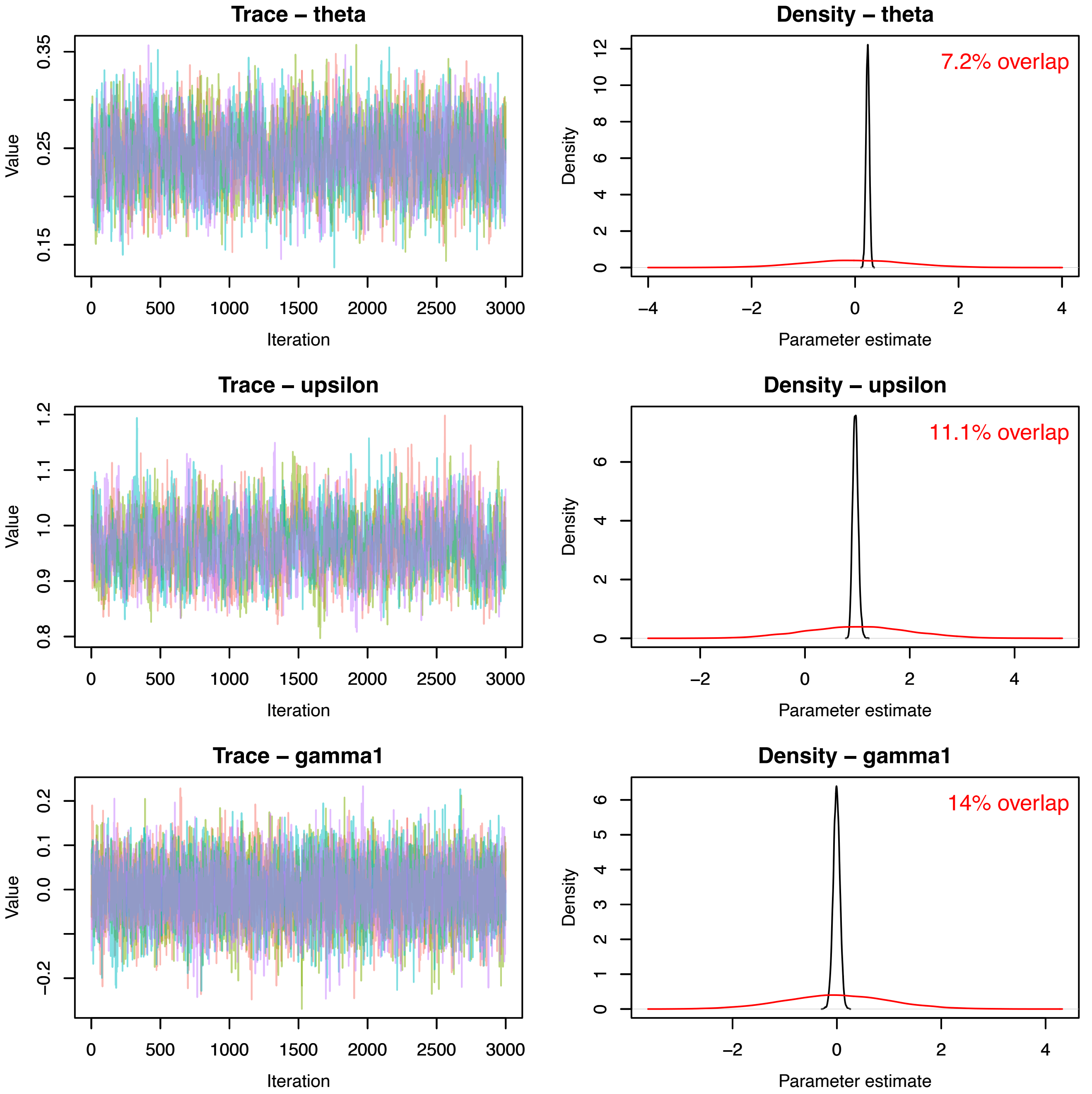

Trace – theta
Value
Iteration
Density – theta
7.2% overlap
Density
Parameter estimate
Trace – upsilon
Value
Iteration
Density – upsilon
11.1% overlap
Density
Parameter estimate
Trace – gamma1
Value
Iteration
Density – gamma1
14% overlap
Density
Parameter estimate

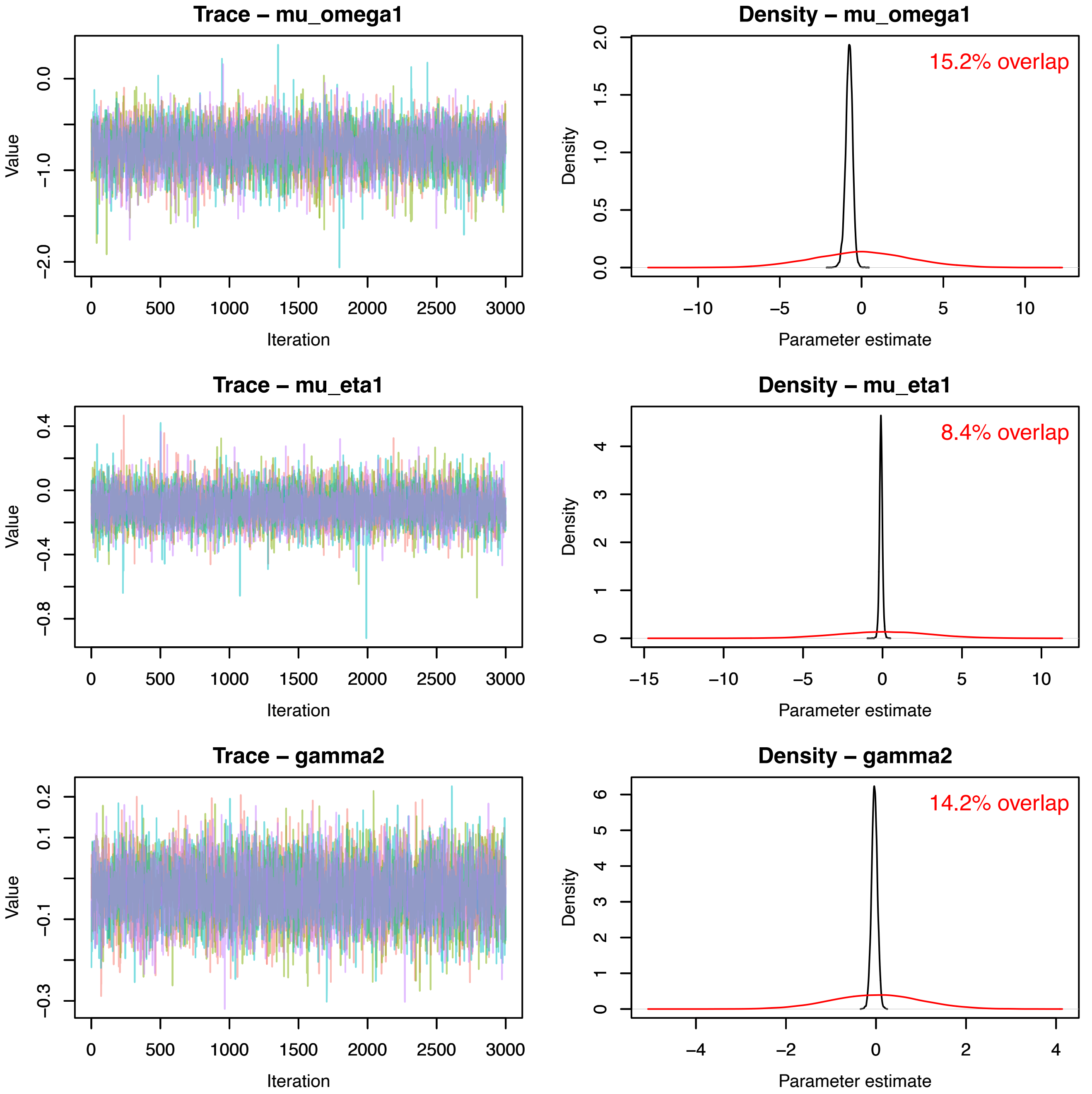

Trace – mu_omega1
Value
Iteration
Density – mu_omega1
15.2% overlap
Density
Parameter estimate
Trace – mu_eta1
Value
Iteration
Density – mu_eta1
8.4% overlap
Density
Parameter estimate
Trace – gamma2
Value
Iteration
Density – gamma2
14.2% overlap
Density
Parameter estimate

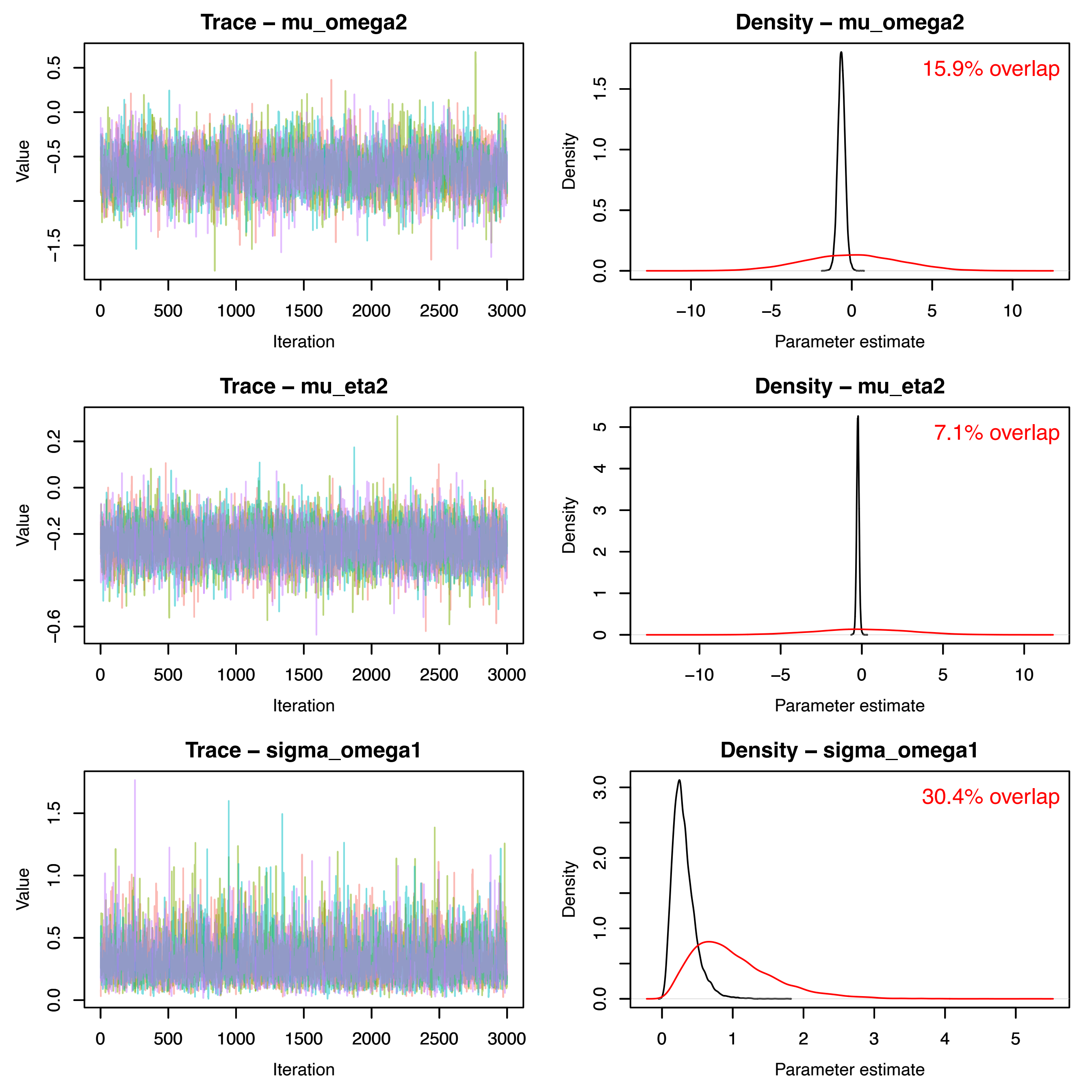

Trace – mu_omega2
Value
Iteration
Density – mu_omega2
15.9% overlap
Density
Parameter estimate
Trace – mu_eta2
Value
Iteration
Density – mu_eta2
7.1% overlap
Density
Parameter estimate
Trace – sigma_omega1
Value
Iteration
Density – sigma_omega1
30.4% overlap
Density
Parameter estimate

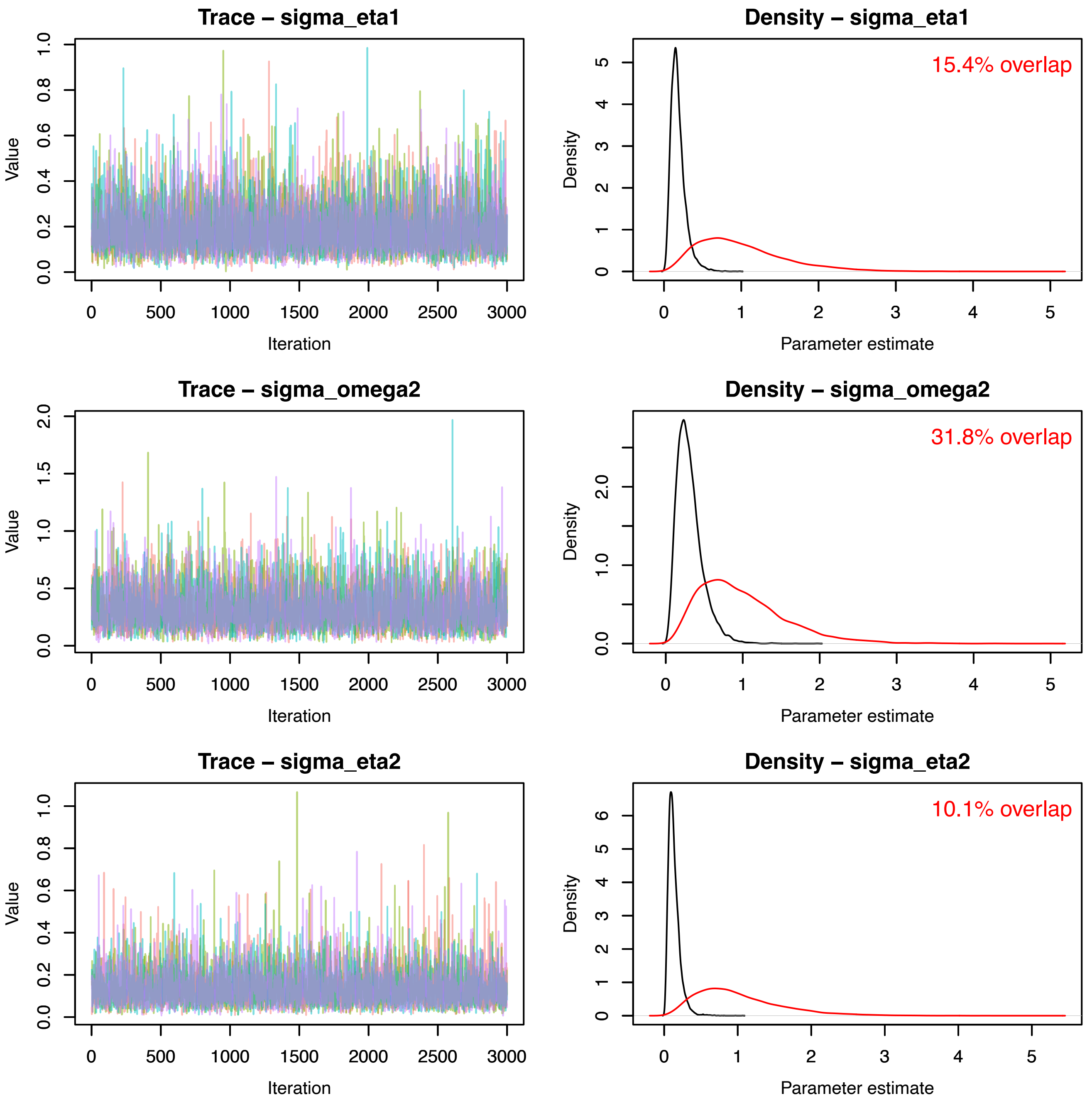

Trace – sigma_eta1
Value
Iteration
Density – sigma_eta1
15.4% overlap
Density
Parameter estimate
Trace – sigma_omega2
Value
Iteration
Density – sigma_omega2
31.8% overlap
Density
Parameter estimate
Trace – sigma_eta2
Value
Iteration
Density – sigma_eta2
10.1% overlap
Density
Parameter estimate

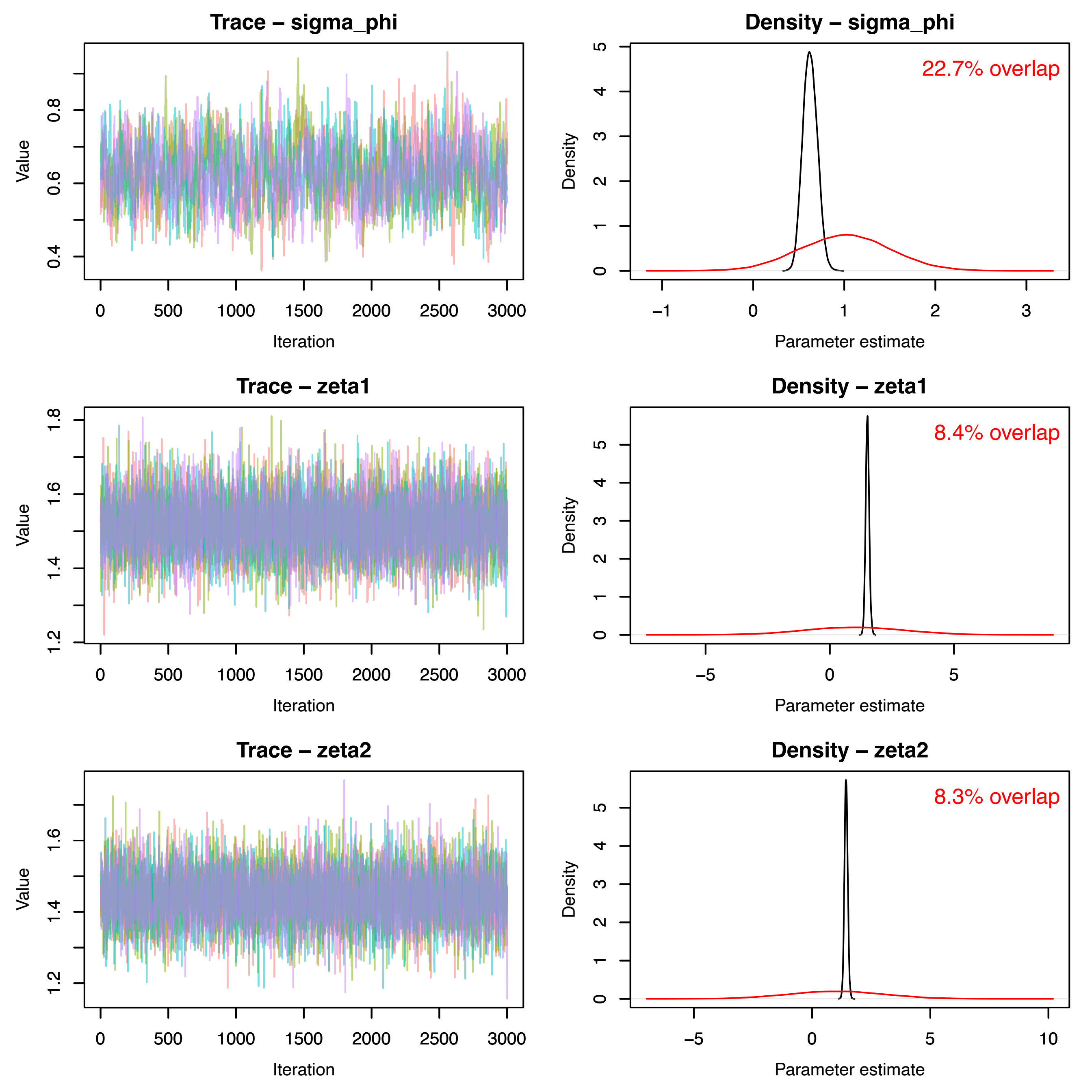
Trace – sigma_phi
Density – sigma_phi
22.7% overlap
Trace – zeta1
Density – zeta1
8.4% overlap
Trace – zeta2
Density – zeta2
8.3% overlap
Value
Iteration
Density
Parameter estimate

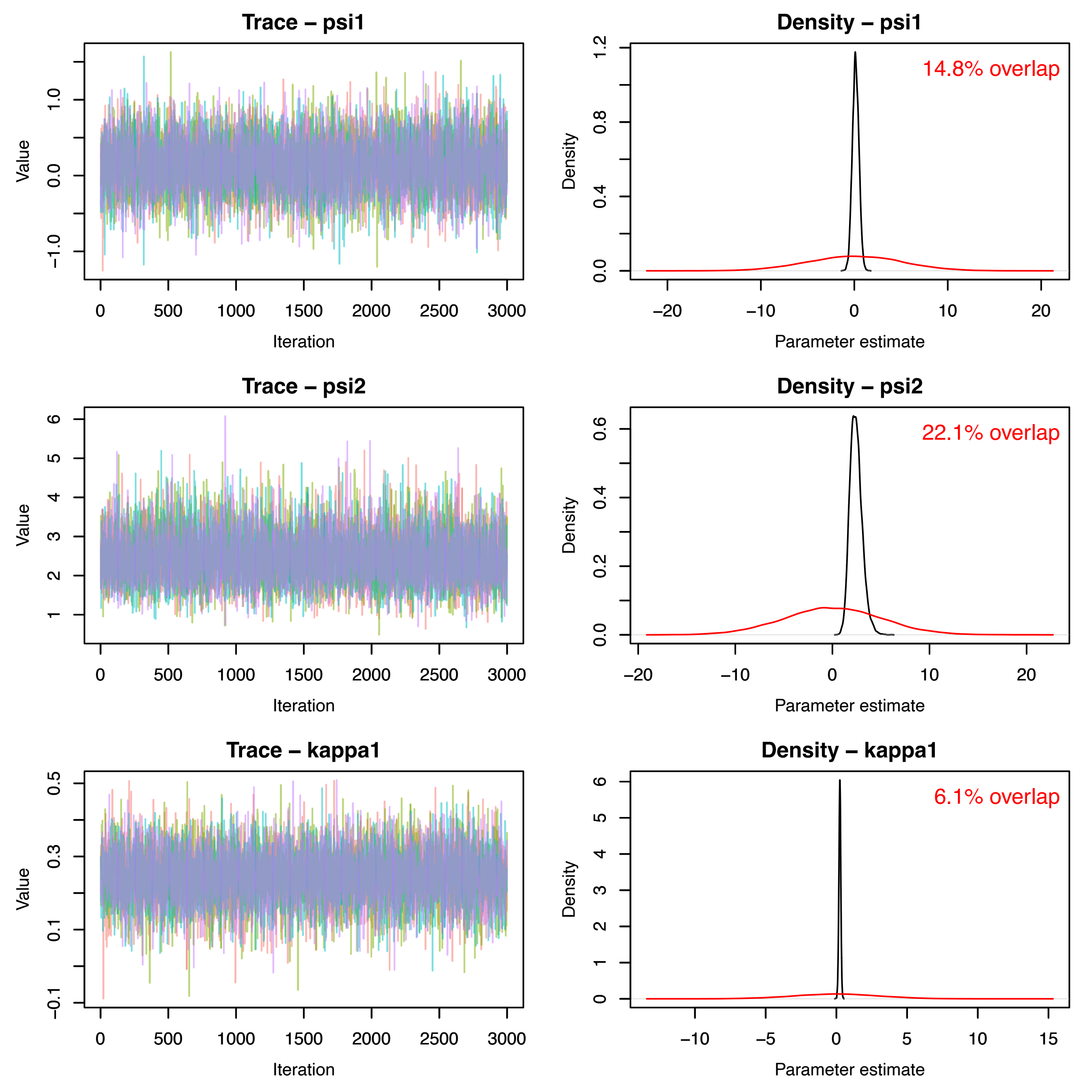

Trace – psi1
Density – psi1
14.8% overlap
Trace – psi2
Density – psi2
22.1% overlap
Trace – kappa1
Density – kappa1
6.1% overlap
Value
Density
Iteration
Parameter estimate

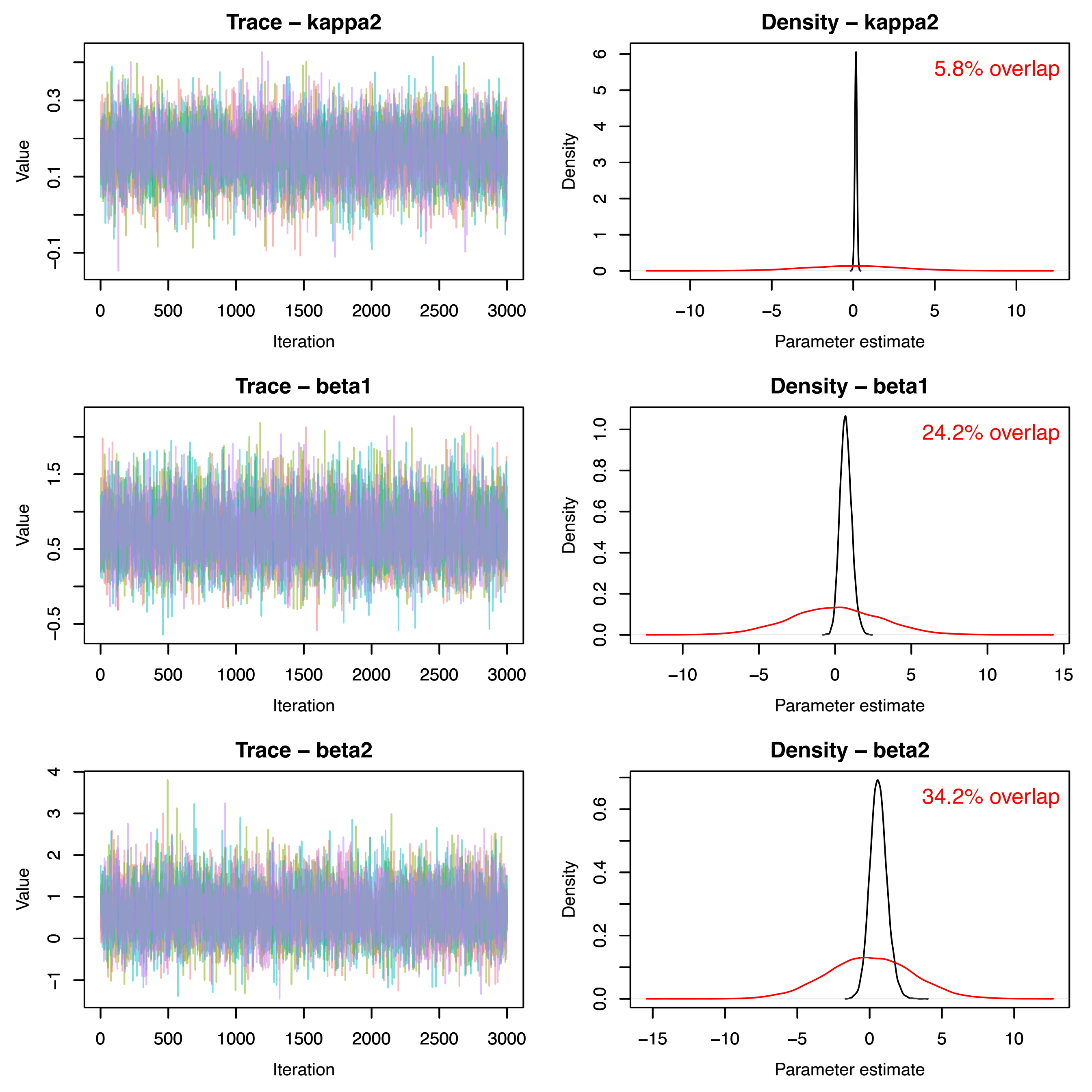

Trace – kappa2
Value
Iteration
Density – kappa2
5.8% overlap
Density
Parameter estimate
Trace – beta1
Value
Iteration
Density – beta1
24.2% overlap
Density
Parameter estimate
Trace – beta2
Value
Iteration
Density – beta2
34.2% overlap
Density
Parameter estimate

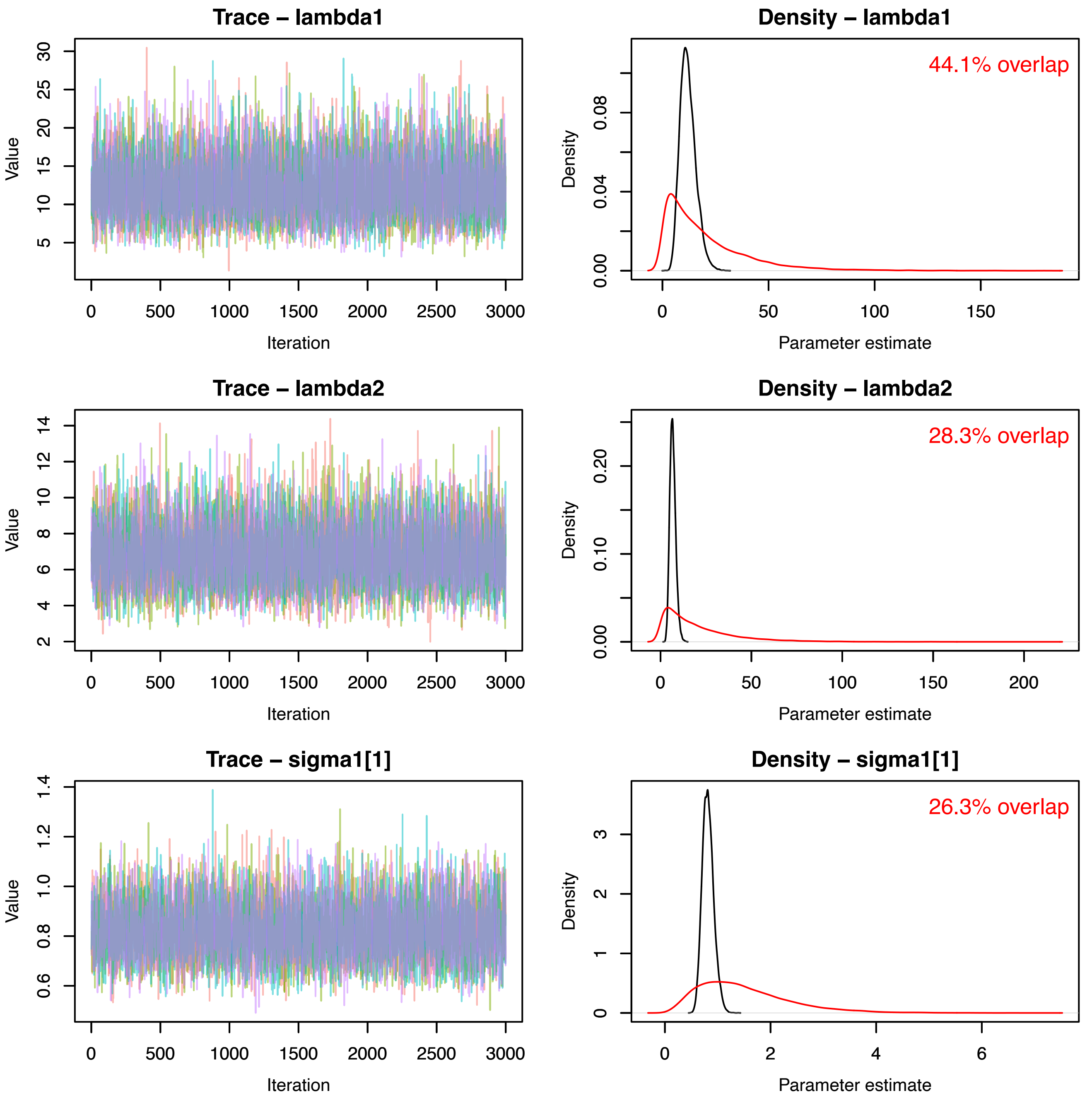
Trace – lambda1
Value
Iteration
Density – lambda1
44.1% overlap
Density
Parameter estimate
Trace – lambda2
Value
Iteration
Density – lambda2
28.3% overlap
Density
Parameter estimate
Trace – sigma1[1]
Value
Iteration
Density – sigma1[1]
26.3% overlap
Density
Parameter estimate

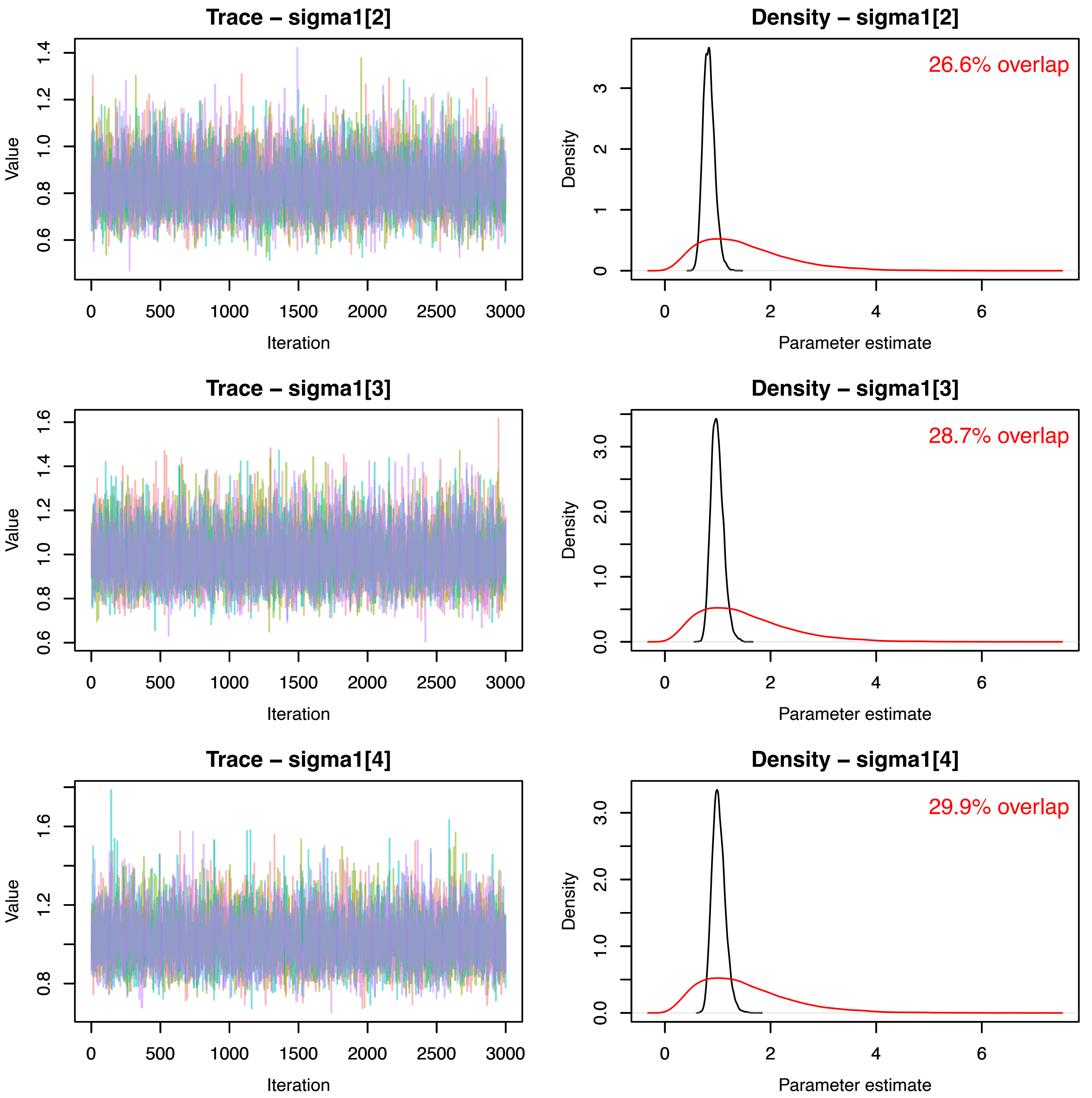
Trace – sigma1[2]
Density – sigma1[2]
26.6% overlap
Trace – sigma1[3]
Density – sigma1[3]
28.7% overlap
Trace – sigma1[4]
Density – sigma1[4]
29.9% overlap
Value
Density
Iteration
Parameter estimate
0
500
1000
1500
2000
2500
3000
2
4
6

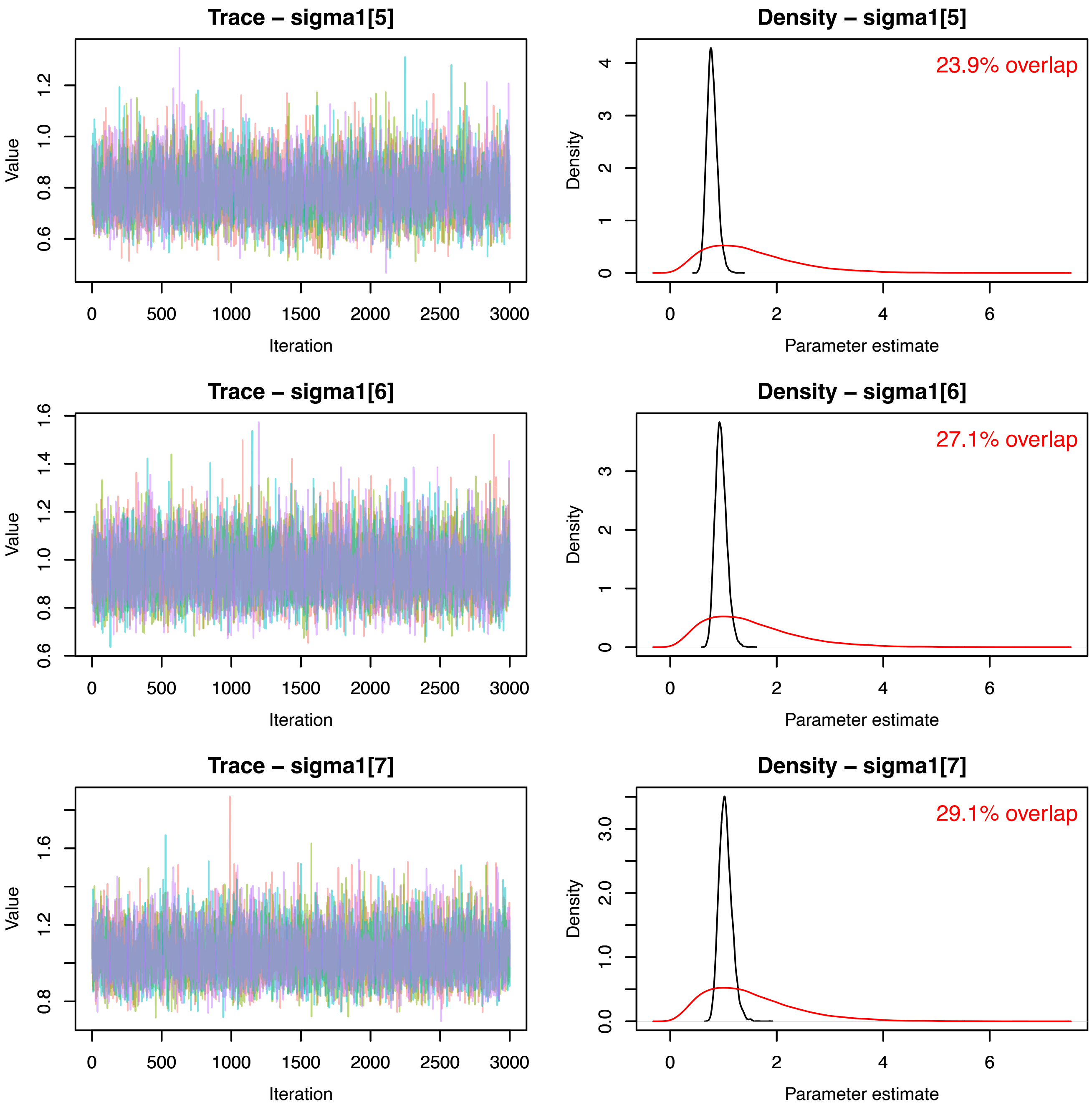
Trace – sigma1[5]
Density – sigma1[5]
23.9% overlap
Trace – sigma1[6]
Density – sigma1[6]
27.1% overlap
Trace – sigma1[7]
Density – sigma1[7]
29.1% overlap
Value
Iteration
Density
Parameter estimate

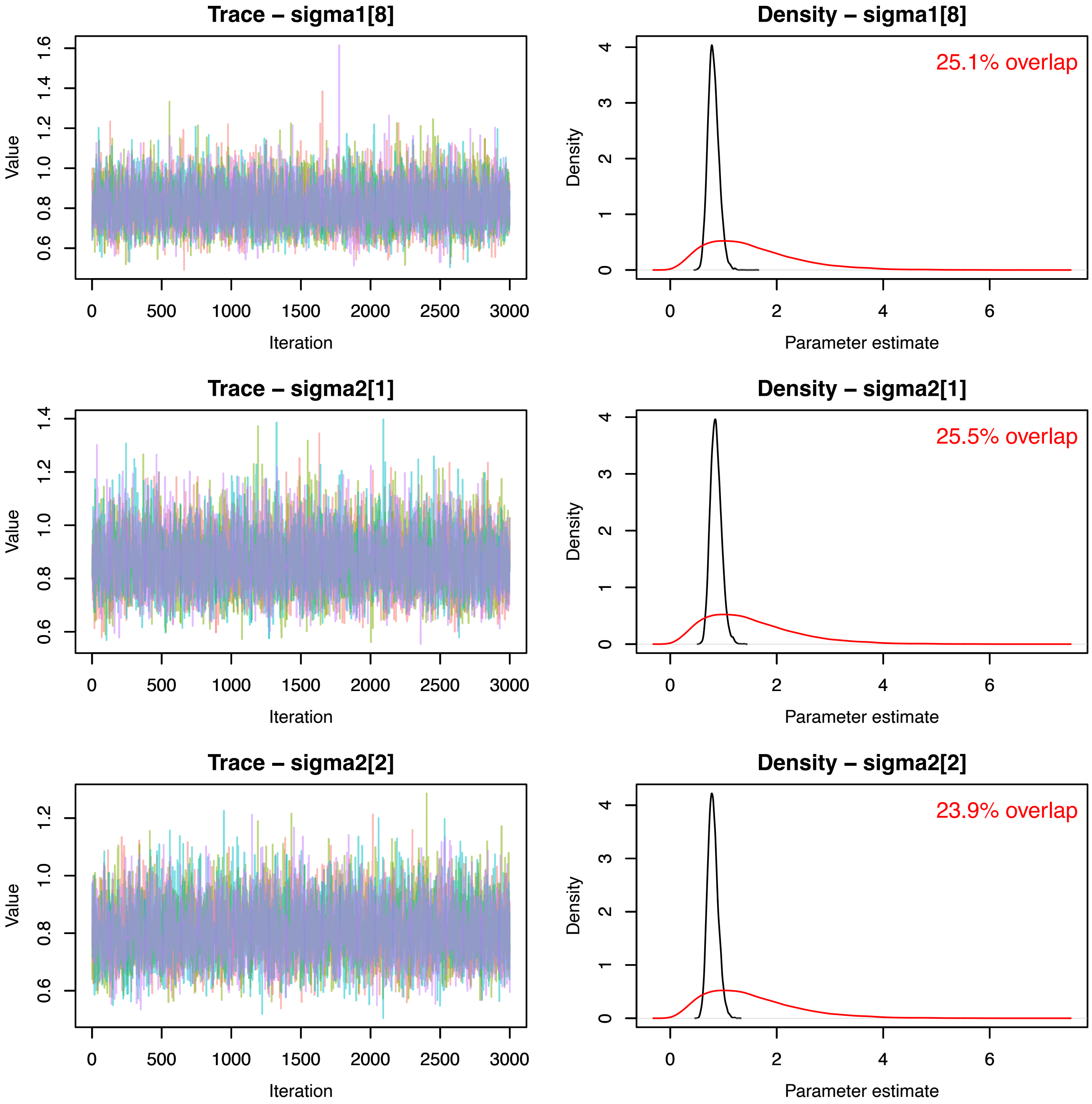
Trace – sigma1[8]
Value
Iteration
0
500
1000
1500
2000
2500
3000
0.6
0.8
1.0
1.2
1.4
1.6
Density – sigma1[8]
25.1% overlap
Density
Parameter estimate
0
2
4
6
0
1
2
3
4
Trace – sigma2[1]
Value
Iteration
0
500
1000
1500
2000
2500
3000
0.6
0.8
1.0
1.2
1.4
Density – sigma2[1]
25.5% overlap
Density
Parameter estimate
0
2
4
6
0
1
2
3
4
Trace – sigma2[2]
Value
Iteration
0
500
1000
1500
2000
2500
3000
0.6
0.8
1.0
1.2
Density – sigma2[2]
23.9% overlap
Density
Parameter estimate
0
2
4
6
0
1
2
3
4

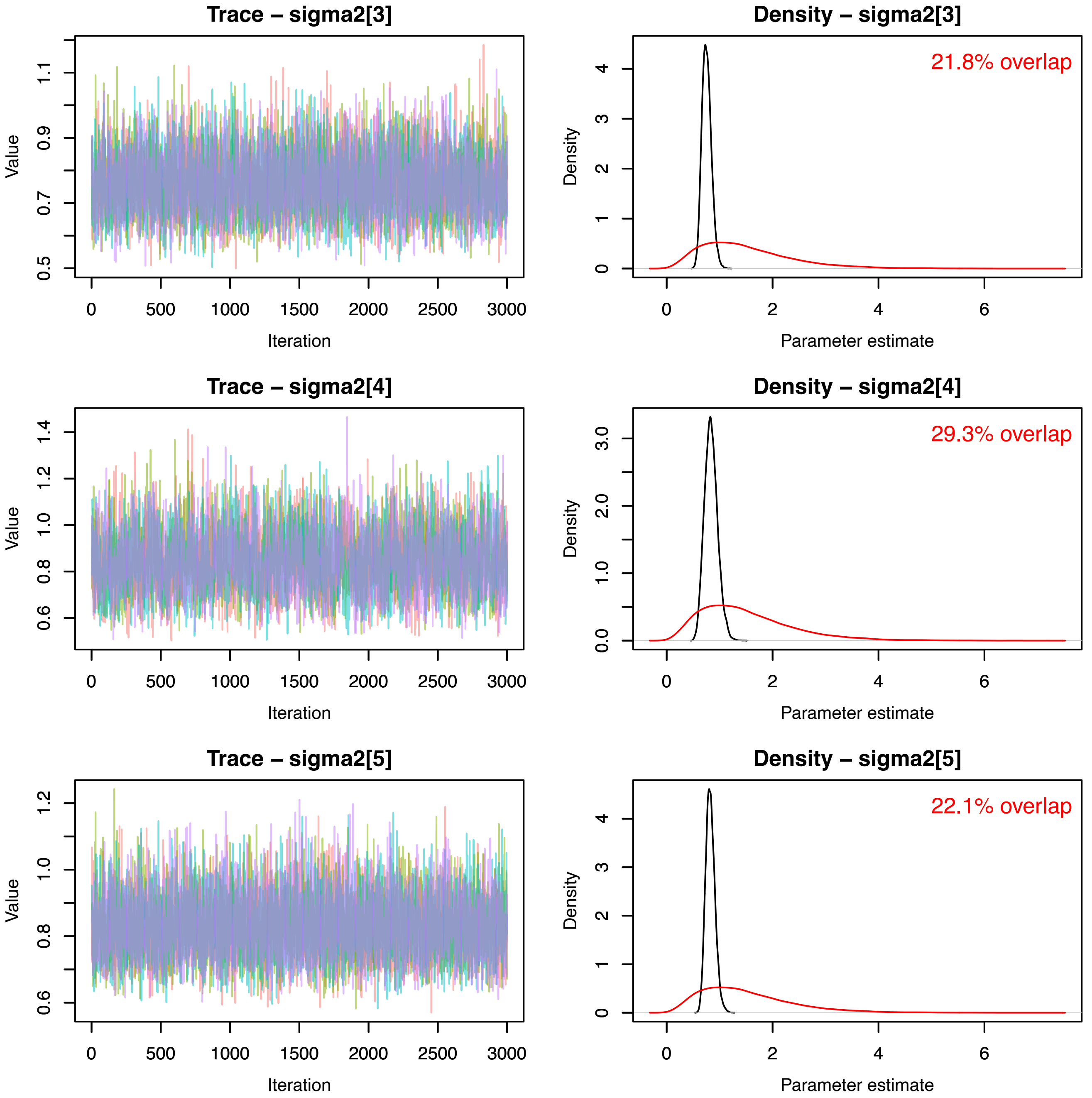
Trace – sigma2[3]
Value
Iteration
Density – sigma2[3]
21.8% overlap
Density
Parameter estimate
Trace – sigma2[4]
Value
Iteration
Density – sigma2[4]
29.3% overlap
Density
Parameter estimate
Trace – sigma2[5]
Value
Iteration
Density – sigma2[5]
22.1% overlap
Density
Parameter estimate

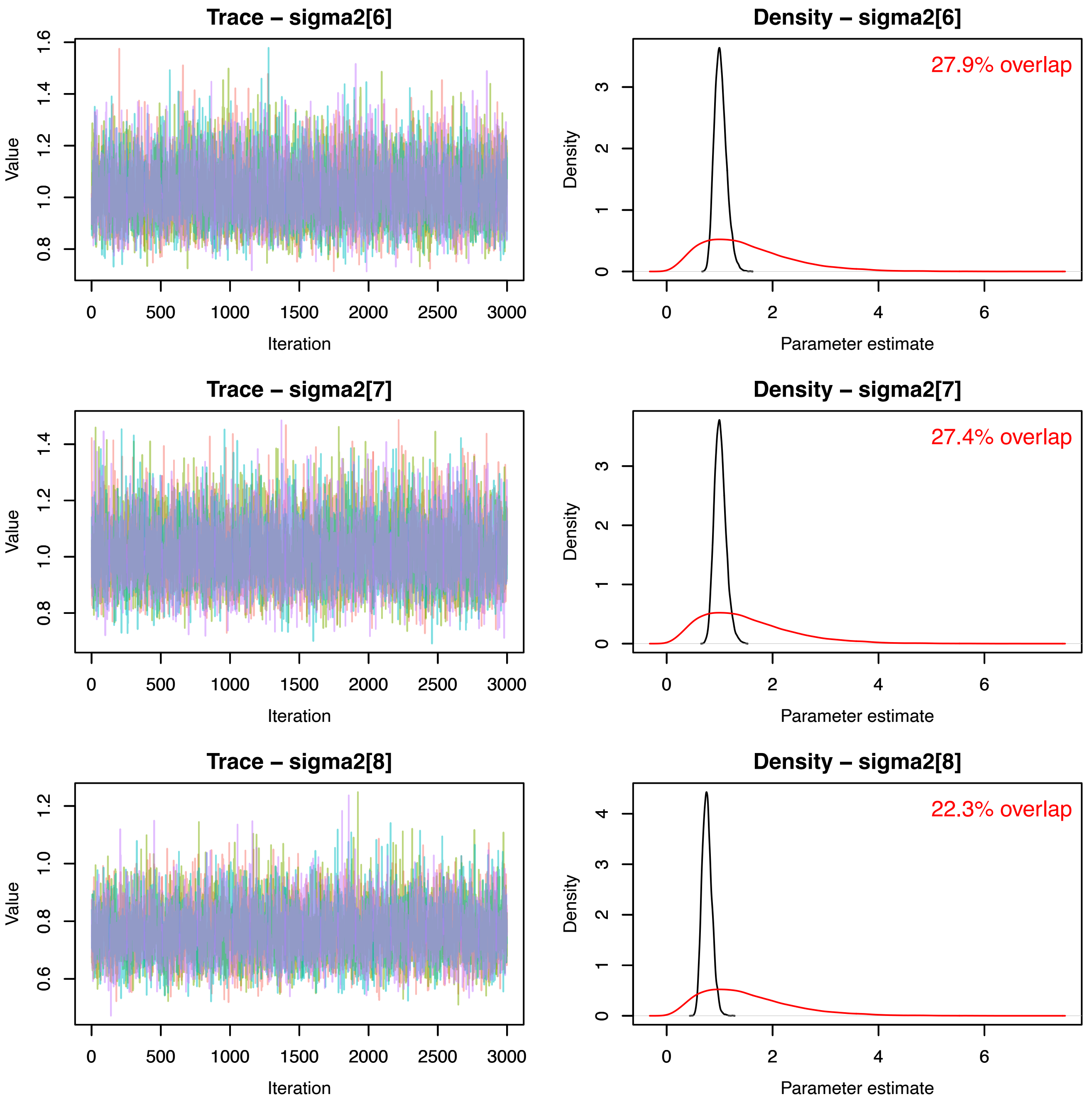
Trace – sigma2[6]
Density – sigma2[6]
27.9% overlap
Trace – sigma2[7]
Density – sigma2[7]
27.4% overlap
Trace – sigma2[8]
Density – sigma2[8]
22.3% overlap
Value
Iteration
Density
Parameter estimate
